# Advances in TCP-Modified PMMA Bone Cements: Relating Microstructure to Mechanical, Biological and Functional Performance—Systematic Review

**DOI:** 10.3390/ma19173772

**Published:** 2026-09-04

**Authors:** Jakub Szabelski, Robert Karpiński

**Affiliations:** 1 Department of Computerization and Production Robotization, Faculty of Mechanical Engineering, Lublin University of Technology, Nadbystrzycka 36, 20-618 Lublin, Poland; 2 Department of Machine Design and Mechatronics, Faculty of Mechanical Engineering, Lublin University of Technology, Nadbystrzycka 36, 20-618 Lublin, Poland; 3 Institute of Medical Sciences, Faculty of Medicine, The John Paul II Catholic University of Lublin, 20-708 Lublin, Poland

**Keywords:** PMMA, bone cement, mechanical properties, osseointegration, bioactivity, setting characteristics, admixtures, ceramics, polymer composites, TCP

## Abstract

The main objective of this systematic review was to synthesise current knowledge on tricalcium phosphate (TCP) as a functional modifier of poly(methyl methacrylate) (PMMA)-based bone cements, relating the microstructure of PMMA/TCP composites to their mechanical, biological and functional (handling) performance. Web of Science, Scopus and PubMed were searched for 2010–2025. Studies reporting primary quantitative data on PMMA cements specifically modified with TCP were eligible. Over one hundred records were screened by two independent reviewers, yielding 15 studies appraised qualitatively and combined by narrative synthesis. The evidence links polymerisation of the PMMA matrix, calcium and phosphate ion release from TCP, apatite-layer precipitation, and cell-mediated TCP resorption coupled to bone remodelling. TCP, especially β-TCP or biphasic calcium phosphate systems, can balance mechanical stability with bioactivity: moderate β-TCP contents (of the order of 10 wt% for solid cements under quasi-static compression) preserve clinically acceptable properties while enhancing osteoconductivity, though this limit falls for porous, α-TCP-containing and fatigue-loaded formulations. Porosity, TCP amount, crystalline form and particle size are the governing microstructural variables. The evidence is limited, dominated by in vitro and short-term static tests, with heterogeneous formulations and no controlled clinical data, so benefits should be interpreted qualitatively rather than as firm quantitative relationships. The review was not registered; this research received no external funding.

## 1. Introduction

Osteoarthritis (OA) is currently one of the most common musculoskeletal disorders, leading to pain, limited mobility and a significant deterioration in quality of life [1,2,3,4,5]. With the progressive ageing of the population, OA is becoming an increasingly significant health and social issue [6]. Despite numerous methods of conservative treatment, including pharmacotherapy, physiotherapy and lifestyle modifications, many patients experience disease progression, resulting in the need for more invasive treatment methods, such as prosthetic replacement [7,8,9]. Prosthetic replacement, i.e., surgical replacement of a damaged joint with a prosthesis, is considered an effective method of treating advanced OA, especially in the case of hip and knee joints [10,11]. The aim of this procedure is not only to relieve pain, but also to restore joint function and improve patients’ quality of life. In recent years, thanks to advances in medical and material technologies, prosthetic replacement has become more advanced, offering better results and longer implant durability.

In clinical practice, both cemented and cementless techniques are used, and the choice of the appropriate method largely depends on the quality of the patient’s bone tissue. Cementless systems require adequate bone density and integrity, which is why their use may be limited or contraindicated in elderly patients with osteoporosis or significant bone loss. The use of bone cement is intended to stabilise and ensure a permanent connection between the prosthesis and the patient’s bone and is one of the key elements in the prosthesis implantation process [12,13,14,15]. Current analyses of the orthopaedic market indicate that polymethyl methacrylate (PMMA)-based bone cements have an almost 60% global share of the bone cement market (material segment), which translates into a dominant frequency of use in cemented procedures. Other biomaterials used for this purpose include calcium phosphate cements, bioactive glasses, hybrid cements, etc. PMMA cements are particularly dominant in hip and knee arthroplasty (where other cements, such as calcium phosphate cements, are rather niche or used as fillers for defects) and in vertebroplasty/kyphoplasty [16,17,18]. Although bone cements have contributed significantly to advances in orthopaedic surgery, their role as potentially the weakest link in the bone–cement–prosthesis system is the subject of intensive research [19,20,21,22,23].

Poly(methyl methacrylate)-based bone cements are a key element of modern orthopaedics and reconstructive surgery thanks to their unique properties, such as biocompatibility, adequate adhesion to bone tissue and adaptability to specific clinical requirements. They are widely used in medicine, mainly in orthopaedics and traumatology. Their key applications include fixing endoprostheses in joint arthroplasty, stabilising and strengthening vertebrae in the treatment of spinal fractures, filling bone defects after tumour resection, stabilising orthopaedic implants and strengthening bones weakened by osteoporosis [24,25,26,27]. PMMA is valued for its strong adhesive properties and compatibility with human tissues, although it carries some risk of complications. However, despite these advantages, there are also challenges associated with its use, particularly in terms of mechanical strength and stability. In response to these challenges, growing research interest is focused on modifying PMMA cements by adding various additives to improve their mechanical, biointegrative and other functional properties, which in turn may contribute to increased durability and clinical effectiveness of implants. The mechanical and functional properties of PMMA-based bone cements depend on many factors related to the preparation process. A key aspect is the mixing method, which affects the homogeneity of the structure and reduces porosity—incorrect mixing can lead to structural defects that weaken the strength of the cement [28,29,30]. Physiological fluids are also important, especially blood and saline solutions during implantation, as well as joint fluid during further use, which may interfere with the polymerisation process and lead to a reduction in mechanical strength and cement adhesion to the bone substrate [31]. Even minor deviations from the typical proportions of components, both monomer and polymer powder, can result in changes in setting time, viscosity and the final mechanical properties of the cement, such as compressive strength and fatigue resistance [32].

Ceramic additives are increasingly used to enhance the performance of bone cements, which is particularly important in the context of their long-term stability and integration with bone tissue. Additives such as hydroxyapatite (HA), calcium phosphates (TCP and CPP), zirconium oxide (ZrO_2_) or titanium dioxide (TiO_2_), and other ceramic and non-ceramic additives can significantly increase the compressive strength of the cement, improve its modulus of elasticity and reduce the degradation process under dynamic loads. Thanks to the bioactivity of some of these materials, cements enriched with ceramic additives exhibit better osseointegration, which promotes implant stability. In addition, ceramics can lower the polymerisation temperature, which reduces the risk of damage to surrounding tissues during cement hardening in clinical conditions. Depending on the type of additives used, it is also possible to increase abrasion resistance and reduce polymerisation shrinkage, which translates into longer durability of bone cements in the body [33,34,35,36,37].

The aim of this article is to review the latest research on the effect of tricalcium phosphate (TCP) on the properties of PMMA-based bone cements and to systematise the current knowledge. The review deliberately covers three complementary property domains that are jointly decisive for clinical performance:

- The biological response and osteogenic potential.
- The mechanical behaviour.
- The handling and processing (functional) characteristics of the cement, together with the underlying microstructure that couples them.

Accordingly, the review addresses the following question: in PMMA-based bone cements, how does the addition of tricalcium phosphate—and its polymorph, content, and particle size—affect the biological response and the osteogenic potential, mechanical behaviour and handling characteristics of a cement, and which microstructural features mediate these effects? Rather than claiming a complete, quantitative composition–microstructure–property map—which the heterogeneity of the available formulations does not yet allow for—this paper aims to organise the evidence across these domains, to make the trade-off between bioactivity and mechanical durability explicit, and to inspire further, more standardised research into implant materials and ways to improve their properties.

## 2. Materials and Methods

This systematic review was conducted and is reported in accordance with the Preferred Reporting Items for Systematic Reviews and Meta-Analyses (PRISMA) 2020 statement. The completed PRISMA 2020 checklist is provided as Supplementary Material, and the flow of records through identification, screening, retrieval and inclusion is presented in Section 3.1. (Characteristics of the Included Studies). This review was not registered. Prospective registration was not undertaken because the principal registers for systematic reviews, including PROSPERO, are restricted to reviews of health-related interventions in human populations and do not accept records for reviews of in vitro and preclinical materials research, which is the subject of the present work. No general-purpose register was used in its place. No formal written protocol was prepared in advance of the review.

The review question, the eligibility criteria, the database selection and the search strategy were specified before the searches were carried out and are reported in full, but they were not deposited in a citable form and cannot be independently verified as pre-specified. The absence of a registered protocol is acknowledged as a limitation of the review process.

Because no protocol was registered or deposited, no amendments to registered information can be reported. Two categories of departure from the original plan should nevertheless be recorded. First, the analytical procedures—the structured appraisal of methodological credibility, the qualitative exploration of the causes of heterogeneity, the sensitivity analyses and the appraisal of the certainty of the evidence—were specified during the preparation of the revised manuscript and after the corpus had been assembled and the primary data extracted. They are therefore post hoc analyses, and although they were applied uniformly across the corpus, they were not pre-specified and should be interpreted accordingly. Second, the classification of the included studies into formulations in which tricalcium phosphate was the principal experimental variable and multi-component formulations was introduced during synthesis, having become necessary once the compositional diversity of the corpus became apparent. The classification was applied to all included studies without exception.

In order to confirm the relevance of the analysed topic, the three most important bibliographic databases (Web of Science, Scopus and PubMed) were reviewed to identify scientific papers dealing with the following topics:

- Bone cements.
- PMMA (polymethyl methacrylate) bone cements.
- TCP-modified PMMA bone cements.

All three databases were searched on the same day and were last searched on 1 February 2026, using the following field-tagged queries, combining the core concepts with Boolean operators and restricting the publication window to the last fifteen years (2010–2025):

*Scopus: (TITLE-ABS-KEY (“bone cement”) AND TITLE-ABS-KEY (pmma) AND TITLE-ABS-KEY (tcp OR “tricalcium phosphate”)) AND PUBYEAR > 2009 AND PUBYEAR < 2026*.

*Web of Science: (All Fields): “bone cement” AND pmma AND (tcp OR “tricalcium phosphate”)*, refined by publication years 2010–2025.

*PubMed: ((“bone cement”[Title/Abstract]) AND (pmma[Title/Abstract])) AND ((tcp[Title/Abstract]) OR (“tricalcium phosphate”[Title/Abstract]))*, refined by publication date from 1 January 2010 to 31 December 2025.

The window was closed at the end of 2025, the last complete publication year at the time of the searches; records published in 2026 were therefore not eligible. No trial or review registers, preprint servers, conference proceedings, thesis repositories or other grey-literature sources were searched; no citation searching of the reference lists of included studies was performed, and no study authors or manufacturers were contacted to identify additional records.

**Figure 1:**
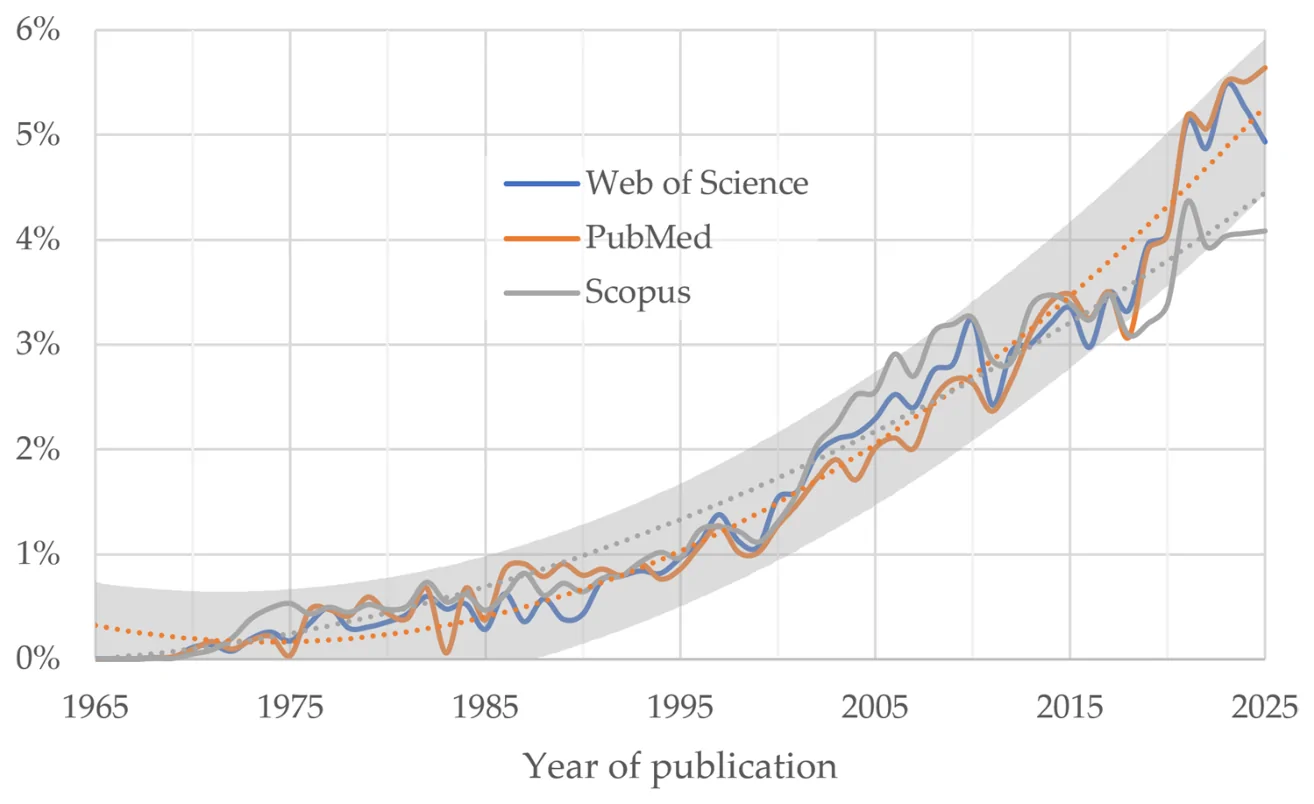
Percentage of total number of papers published on “bone cements” over years.

Research into bone cements began in the mid-1960s. Due to the varying number of studies in different databases, 100% of the studies from each database and the distribution of their publication dates were analysed (Figure 1). As can be seen, it was not until around 2000 that there was a significant increase in the number of studies dealing with this biomaterial, which is related to its widespread use in medical practice. The last 18–19 years account for a total of 2/3 of all publications (depending on the database).

**Figure 2:**
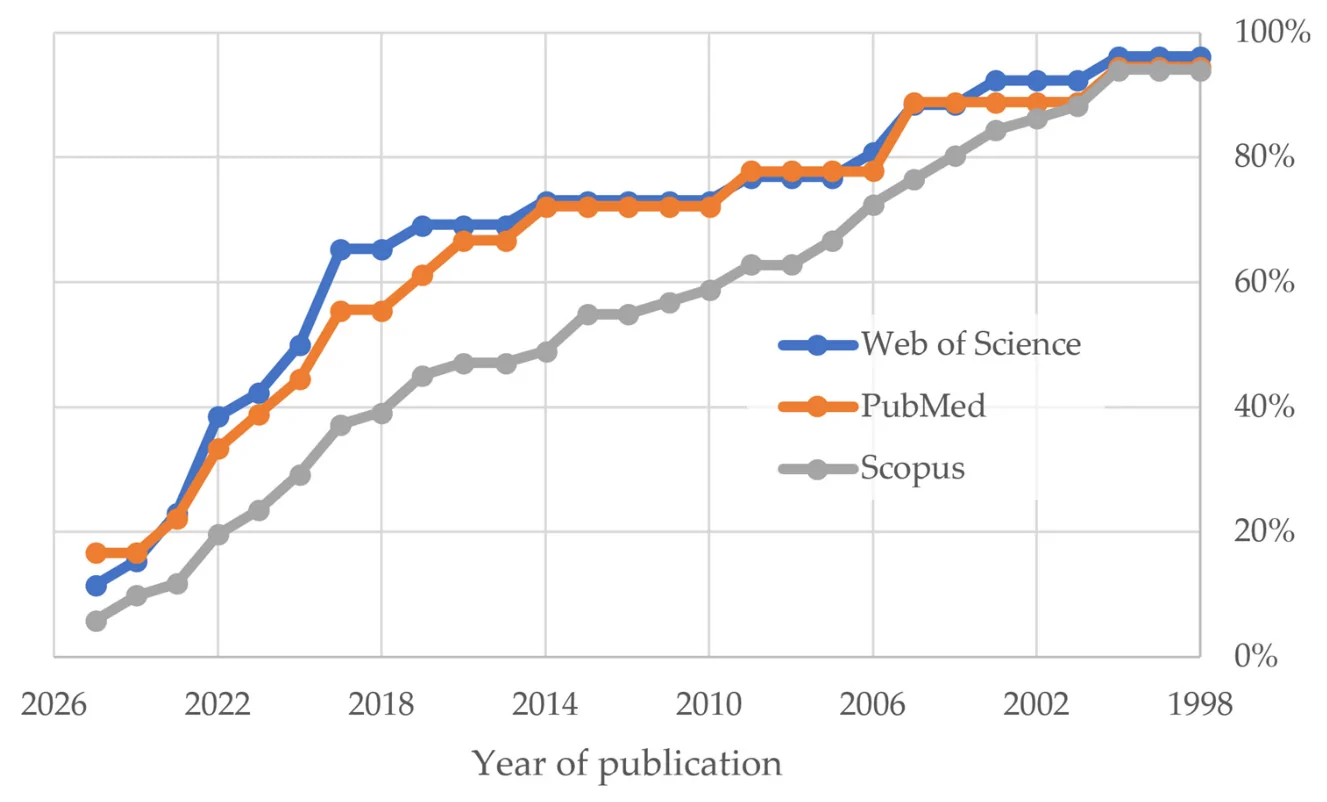
Cumulative share of research papers on “bone cement” AND pmma AND (tcp OR “tricalcium phosphate”) by publication year.

However, half of all publications in this area are no older than 12–15 years. In the case of the analysis of the occurrence of the summing phrase “bone cement AND pmma”, a period of 15 years covers 2/3 of all published research results. An analysis of the most detailed phrase, “bone cement AND pmma AND (tcp OR “tricalcium phosphate”)”, shows that the topic of adding tricalcium phosphate to bone cements is still relevant and that the research being conducted is published on an ongoing basis (Figure 2).

A preliminary review found that a significant proportion of records would be duplicated in the analysed databases, so a cut-off date of the last 15 years was finally adopted, covering approximately two-thirds of all works from the Web of Science and PubMed databases and approximately 57% of works from Scopus. On this basis, a fifteen-year window was adopted, and the identification, screening and inclusion of records were carried out and are reported according to the PRISMA 2020 statement [38]. The resulting flow of records is presented in Section 3.1. (Characteristics of the Included Studies). The review concerned selected phrases that were searched for in titles, abstracts and keywords. In the next steps, the initial 105 records obtained from the database review were filtered by removing duplicate works between databases and articles that did not meet the publication time criterion. The remaining works were obtained in PDF format where possible. For reports not available through institutional subscriptions or publisher open-access platforms, additional lawful retrieval procedures were undertaken. These included searches for author-shared full texts and accepted manuscripts through ResearchGate, an academic networking platform that permits authors to share eligible versions of their work and enables readers to request full texts directly from authors. Where available, author-provided accepted manuscripts—i.e., post-peer-review versions not yet publisher-copy-edited or typeset—were used for eligibility assessment and data extraction. Reports for which no sufficiently complete full text could be obtained after these procedures were classified as not retrieved.

The collected works were catalogued using the open-source software Zotero (2025), which enables effective analysis, browsing and grouping of articles, significantly streamlining research work.

The analysis of the works was not limited for linguistic reasons, even though the vast majority of the works were in English, with a few in other languages (Chinese, Brazilian Portuguese). Modern language tools (translation tools) almost completely eliminate language barriers today. They combine machine learning models with deep learning and use neural networks to process entire sentences along with their context. Thanks to the fact that they are trained on millions of translated texts, it is possible to translate even specialised technical and medical texts naturally and accurately. At this stage, several articles were also rejected which, despite meeting the assumed keywords in the abstract or title, did not directly address the analysed problem in their content, e.g., due to only a substantive reference to PMMA cements—they focused on research into other types of cements.

The eligibility of records was governed by explicit inclusion and exclusion criteria, applied sequentially during title/abstract screening and full-text assessment. A study was included when it met all of the following conditions:

- It investigated PMMA-based bone cement specifically modified with tricalcium phosphate (α-TCP, β-TCP or biphasic calcium-phosphate systems containing TCP), rather than calcium-phosphate or other ceramic cements in general;
- It reported original, quantitative experimental data on at least one of the property domains relevant to this review-mechanical (e.g., compressive/flexural strength, modulus), biological (e.g., osseointegration, bioresorption, cytotoxicity) or handling/functional (e.g., setting time, polymerisation temperature, wettability);
- It was published within the adopted fifteen-year window (2010–2025), which already covers approximately two-thirds of the entire literature on PMMA bone cements;
- Its full text was retrievable and written in a language that could be reliably processed, including via contemporary machine-translation tools.

Conversely, records were excluded when any of the following applied:

- The target keywords appeared only incidentally in the title or abstract while the study did not directly concern PMMA/TCP cements-for example, works in which PMMA or TCP were merely cited as examples of other cement systems;
- The record was a duplicate across the three databases;
- The study was published before 2010 and therefore outside the adopted time window;
- The full text could not be retrieved (typically because it was behind a paywall);
- The record did not report primary experimental data (e.g., conference abstracts, editorials or purely narrative notes).

Because the number of eligible records was small, screening did not rely on an automated tool: the pre-screening corpus was examined manually, record by record, and papers were eliminated against the criteria above. Two reviewers independently appraised each candidate study, and discrepancies were resolved by discussion until consensus was reached.

Data were extracted from the full text of each included report into a purpose-built extraction form corresponding to the columns of Tables 1 and 2. Both authors extracted data from every included report independently, and the two datasets were compared entry by entry. Discrepancies were resolved by returning to the source publication and by consensus. No automation tools were used for data extraction, study investigators were not contacted to obtain or confirm missing data, and values that the original authors reported only graphically were read from the published figures and are marked as approximate (≈) in Tables 1 and 2.

Data were sought for outcomes in the three property domains that define the scope of this review. In the mechanical domain: quasi-static compressive strength and flexural (three-point bending) strength, determined according to ISO 5833 [39] or ASTM F451 [40] where the original authors invoked those standards, flexural modulus, Young’s modulus and nanoindentation modulus, hardness (Shore D or microhardness), the strength of the cement–bone interface however operationalised by the original authors (bone bonding or detaching strength), the shear strength of the cement-metal interface, screw pull-out force and deformation of specimens conditioned in liquid. In the biological domain: osteointegration, quantified as the affinity index (the proportion of the cement surface in direct contact with bone), the new-bone volume fraction (BV/TV), the depth of bone ingrowth into the cement, the proportion of the defect bridged by bone, and semi-quantitative histological osteointegration grades, cell viability and cytotoxicity in the cell lines used by the original authors, alkaline phosphatase activity and mineral deposition as markers of osteogenic differentiation, degradation expressed as mass loss, release of incorporated agents, antibacterial activity against the strains tested and the qualitative character of the cement-bone interface, that is direct bone apposition versus an interposed fibrous layer. In the handling and functional domain: setting and working time, peak polymerisation temperature, injectability, water uptake, and surface wettability.

Data were additionally sought, but were not found in any included study, for fatigue strength or fatigue life, creep and stress relaxation, fracture toughness, hydrolytic ageing beyond the follow-up periods reported, and any patient-relevant clinical endpoint, including implant survival, revision rate, clinically confirmed thermal injury and patient-reported outcomes. The absence of these data is reported as a finding of this review and constrains the certainty of its conclusions.

For every included study, all results compatible with the outcomes listed above were sought and extracted, for every formulation, specimen condition, loading mode and time point reported. No subset of results was selected on the basis of statistical significance or of the direction of effect. Where a study reported a series of compositions, the entire series was extracted, so that dose–response behaviour rather than the single most favourable composition could be assessed. Where an outcome was reported at more than one time point, all time points were extracted and tabulated in Table 2, and the longest available follow-up was used in the cross-study comparison. Where the same outcome was reported both numerically and graphically, the numerical value was used. Outcomes were retained in the units used by the original authors, without conversion between measurement systems.

For each included study the following study-level variables were sought and, where reported, extracted: the PMMA base cement or experimental system, identified by commercial name and manufacturer where stated, the TCP polymorph (α-TCP, β-TCP, a biphasic calcium phosphate containing TCP, or a doped TCP phase), together with any co-additives and their contents; the nominal TCP content and the basis on which the original authors expressed it, the TCP particle or granule size and, for porous formulations, the pore size and pore-size distribution, total porosity and any reported degradation or mass loss, the mechanical test methods, including the standard invoked and, where stated, specimen geometry, conditioning medium and loading rate; the study type (in vitro, ex vivo, in vivo, or a combination), the biological model, species, defect site and follow-up duration; the comparator or control against which the modified formulation was assessed and the sample size per experimental group. These variables constitute the columns of Table 1. The funding sources and declared conflicts of interest of the included studies were not extracted.

Variables that a report did not provide are recorded as not reported (“NR”) and were neither imputed nor estimated nor inferred from related publications by the same authors. Four specific assumptions were made. First, nominal TCP contents were recorded exactly as expressed by the original authors and were not recalculated to a common basis; where content is given as a fraction of the powder phase, this is identified in Table 3, and such values are not directly comparable with contents expressed relative to the whole set cement. Second, where the TCP polymorph was not specified, the study is recorded as containing TCP of unspecified phase and was not assumed to contain β-TCP. Third, sample sizes are recorded on the basis used by the original authors, whether per experimental group, per animal or per anatomical site, without conversion between these bases. Fourth, where porosity or degradation was characterised only by microscopy without a numerical value, it is recorded as qualitative and was not converted into a numerical estimate. Quantitative values that the original authors presented only in graphical form were read from the published figures and are marked as approximate (≈) in Tables 1 and 2. Where a report presented outcomes obtained in the authors’ own earlier publications rather than in the report itself, the provenance of those data is indicated in Table 1, and they were not treated as original findings of the included report.

Three syntheses were planned, corresponding to the three property domains defining the scope of this review and presented in separate sections. A study was eligible for a given synthesis if it reported at least one of the outcomes listed for that domain. A single study could therefore contribute to one, two or all three syntheses, and the syntheses are not based on mutually exclusive subsets of the corpus.

Assignment was made by tabulating the extracted formulation, design and outcome characteristics of each included study and comparing them against the planned groups, rather than by judgement made during the writing of each section. Results of individual studies are displayed in tabular form: study characteristics in Table 1 and extracted outcome values in Table 2, with values read from published figures marked as approximate. The three evidence syntheses are summarised graphically as a bioactivity–mechanical-stability trade-off across the reviewed literature. No forest plots or other meta-analytical graphics were produced, since no statistical synthesis was undertaken.

Within each synthesis, studies were further assigned to two pre-specified formulation classes: class I, in which tricalcium phosphate was the principal experimental variable and was compared against an otherwise equivalent unmodified PMMA control, and class II, multi-component formulations in which TCP was combined with further modifiers such as HA or biphasic calcium phosphate, HEMA, chitosan, bioactive glass or silver-, selenium- or copper-containing phases. Assignment to class I required that the source publication reported at least one pair of formulations differing only in TCP polymorph or TCP content. Only class I studies were used to support conclusions attributed to TCP itself. Findings from class II studies are reported as properties of the complete formulation. Where a study contained both class I and class II comparisons, the relevant comparisons were assigned separately rather than the study as a whole.

Studies that reported an outcome only as a direction of change, without numerical values, were retained in the corresponding synthesis but are identified as qualitative contributions in Table 2 and were not used to establish quantitative thresholds. Studies reporting no outcome in a given domain were not included in that synthesis, and this is stated explicitly rather than left to be inferred from the absence of a citation.

Results were combined by structured narrative synthesis, reported in accordance with the SWiM (Synthesis Without Meta-analysis) guidance. Meta-analysis was neither planned nor attempted. The included studies differ systematically in PMMA base cement, TCP polymorph, content and particle size, co-additives, porosity and pore architecture, specimen geometry, conditioning medium and loading protocol, and biological model and follow-up duration. Within each synthesis, findings were ordered by formulation class and study design, and the direction and magnitude of each effect were described relative to the comparator of the same study.

Possible causes of variation between study results were examined qualitatively by comparing outcomes across subgroups defined in advance: TCP polymorph (α-TCP, β-TCP, or a biphasic calcium phosphate containing TCP), nominal TCP content, particle-size class (fine powder versus granules of 100–300 µm), solid versus deliberately porous architecture, the presence and type of co-additives and loading mode (quasi-static versus cyclic). Where two studies reported effects of opposite direction for the same nominal outcome, the discrepancy was examined against these subgroups before any interpretation was drawn. Formal subgroup statistics and meta-regression were not applicable, since no quantitative synthesis was performed and the number of comparable estimates within any subgroup was too small to support them.

Because the syntheses were narrative, robustness was assessed by repeating each synthesis with defined subsets of the corpus set aside and determining whether the direction and the stated magnitude of the principal findings were preserved. Four sensitivity analyses were specified. First, reports that present previously published experimental data rather than original measurements were set aside to establish whether any conclusion depended on evidence reported at second hand. Second, studies in which a tricalcium phosphate cement was evaluated alongside, rather than incorporated into, a PMMA matrix were set aside to establish whether the conclusions concerning TCP-modified PMMA were affected by the presence of such comparative studies in the corpus. Third, a study co-authored by the present authors was set aside to establish which findings remain supported by independent evidence alone. Fourth, quantitative values read from published figures were set aside in favour of numerically reported values only. In each case, a finding was regarded as robust if its direction was unchanged and its reported magnitude remained within the range spanned by the remaining studies.

Given the marked diversity of study designs among the included evidence, including in vitro materials studies, cell-culture experiments, and in vivo animal investigations, a single formal, instrument-based risk-of-bias assessment, such as SYRCLE or ROBINS tools, was not considered applicable across all study designs. The methodological credibility of the included works was therefore evaluated using a structured qualitative appraisal. This appraisal considered the completeness of reported data, sample size where reported, the presence of appropriate control or comparator groups, the reported mechanical test methods and reference to recognised standards (e.g., ISO 5833 and ASTM F451), the biological model, follow-up duration, and major methodological limitations relevant to interpretation. These characteristics are summarised within Tables 1 and 2. The absence of a standardised quantitative quality-scoring instrument is acknowledged as a limitation of the present review.

No pooled effect measures were computed, since no meta-analysis or other statistical synthesis was undertaken. For the presentation and comparison of results, the following effect measures were used. For continuous mechanical, thermal and handling outcomes, results are reported as the absolute values given by the original authors, with the measure of dispersion they provided (mean ± standard deviation where available), together with the change relative to the unmodified PMMA comparator of the same study, expressed as a percentage of that comparator. Relative changes were computed from the reported group means only. They are descriptive quantities and are not accompanied by confidence intervals, and they are not reported where the source publication did not provide a comparator measured under identical conditions. For biological outcomes, results are reported in the units used by the original authors—the affinity index and the proportion of the defect bridged by bone as percentages, the new-bone volume fraction as BV/TV in percent, interface strength in the units reported, contact angle in degrees—with the direction of effect stated relative to that study’s own control. Where a study assessed osteointegration on a semi-quantitative histological scale, the reported grades are given as such and were not converted into a numerical effect measure or treated as an interval scale.

Where a report provided only a statement of statistical significance without an effect size, the reported *p*-value and its threshold are quoted, and no effect measure was derived. Where a report described only the direction of change without numerical values, the direction is reported as such and is identified in Table 2 as a qualitative finding.

In addition to within-study comparisons, absolute mechanical values were compared against the minimum requirements of ISO 5833 for compressive strength, flexural strength and flexural modulus. This comparison uses the standard, rather than an unmodified control, as the reference, and is reported separately from the within-study relative changes.

Statistical methods for detecting reporting bias, including funnel-plot asymmetry and its formal tests, were not applicable, since no quantitative synthesis was performed and no outcome was reported by a sufficient number of comparable studies. Prospective protocol registration is not established practice for in vitro and preclinical materials research, so reported outcomes could not be compared against pre-registered ones. Risk of bias due to missing results was therefore assessed in two ways. First, within each included report, the outcomes named in its methods section were compared against those presented in its results, and any outcome measured but not reported, or reported without the corresponding numerical value or measure of dispersion, was recorded in the final column of Tables 1 and 2. Second, at the level of the corpus, the number of reports identified as potentially eligible but not obtainable in full text was recorded, and the potential consequences for each synthesis were considered. No attempt was made to obtain unreported values by contacting the original authors, and this is acknowledged as a limitation.

The certainty of the body of evidence was appraised for each of the three syntheses and for each principal finding within them. Formal GRADE assessment was not applied because several of its domains, and in particular imprecision and indirectness as defined for clinical outcomes, are not transferable without modification to a body of evidence composed of in vitro materials tests, cell-culture experiments and short-term small-animal studies containing no patient-relevant endpoint. Certainty was therefore appraised descriptively, by both authors and by consensus, against four considerations applied uniformly to every finding: first, the consistency of the direction of effect across independent studies; second, the number of studies supporting the finding, a finding resting on a single report being treated as provisional irrespective of the quality of that report; third, the extent to which tricalcium phosphate was the isolated experimental variable, findings obtained in multi-component formulations or under simultaneously varying powder-to-monomer ratios being regarded as weaker evidence of attribution; and fourth, the completeness of the underlying reporting, that is whether the effect was supported by numerical values with a measure of dispersion or only by a stated direction of change. On this basis, each finding was classified as being moderate certainty, where the direction of effect was consistent across independent studies and adequately reported, or of limited certainty, where it rested on a single study, on incompletely reported outcomes, or on formulations in which the contribution of TCP could not be separated from that of co-additives. No finding was classified as of high certainty, since the corpus is entirely preclinical, is dominated by short-term in vitro and small-animal work, and contains no clinical endpoint; the two-level classification therefore reflects the ceiling imposed by the available evidence rather than a decision to compress a finer scale. The resulting appraisals are reported in the text at the close of each synthesis rather than in a separate summary-of-findings table, the number of distinct findings being small enough for them to be stated in full where the corresponding evidence is presented.

## 3. The Effect of Admixtures on the Properties of Bone Cements

The introduction of ceramic additives to PMMA bone cements is meant to improve their mechanical, biological and functional properties. Ceramic additives can increase the compressive strength, flexural strength and fatigue resistance of PMMA cements. Ceramic particles act as a reinforcement for the cement structure, limiting the propagation of microcracks and improving its durability under dynamic loads.

Some additives, such as hydroxyapatite (HA) or calcium phosphates (TCP and CPP), exhibit bioactivity, which means that they can promote the formation of new bone tissue in contact with the cement. This improves the osseointegration of implants, increasing their stability and reducing the risk of loosening. The introduction of bioactive components can also modulate the biological response, reducing the risk of inflammatory reactions. The addition of TCP powder can also act as a bioactive carrier, e.g., for other biomaterials [41].

As PMMA cements are known for their exothermic polymerisation reaction, which can lead to local tissue overheating, the incorporation of ceramic additives can also modify the thermal profile of curing. One of the problems with acrylate cements is also polymerisation shrinkage, which can lead to internal stresses and microcracks. The introduction of ceramic particles with a suitable structure can reduce this effect, improving the dimensional stability of the cement after curing. The addition of ceramic admixtures can affect the viscosity of the cement, its setting time and its ability to penetrate the bone structure [42,43,44]. Optimising these parameters is crucial for obtaining the right application properties and facilitating the work of surgeons during implant procedures.

Despite its many advantages, the use of ceramic additives requires careful selection of their type, particle size and concentration in order to avoid negative effects such as excessive increase in cement brittleness or hindrance to the mixing process. It is also important to ensure adequate compatibility between the polymer and ceramic phases to avoid phase separation and adverse effects on the cement microstructure. It is worth noting that the effect of ceramic additives on PMMA bone cements varies and depends on the type and amount of the additive. Further research is needed to better understand these relationships and optimise the composition of bone cements for specific orthopaedic and dental applications [45,46,47]. The thematic division of analyses adopted below is purely conventional and serves to organise the available data, as individual modifications may simultaneously affect many characteristics of the material and aspects of its use. Therefore, this classification does not necessarily exhaust the full range of interphase interactions occurring in modified cement systems.

### 3.1. Characteristics of the Included Studies

**Figure 3:**
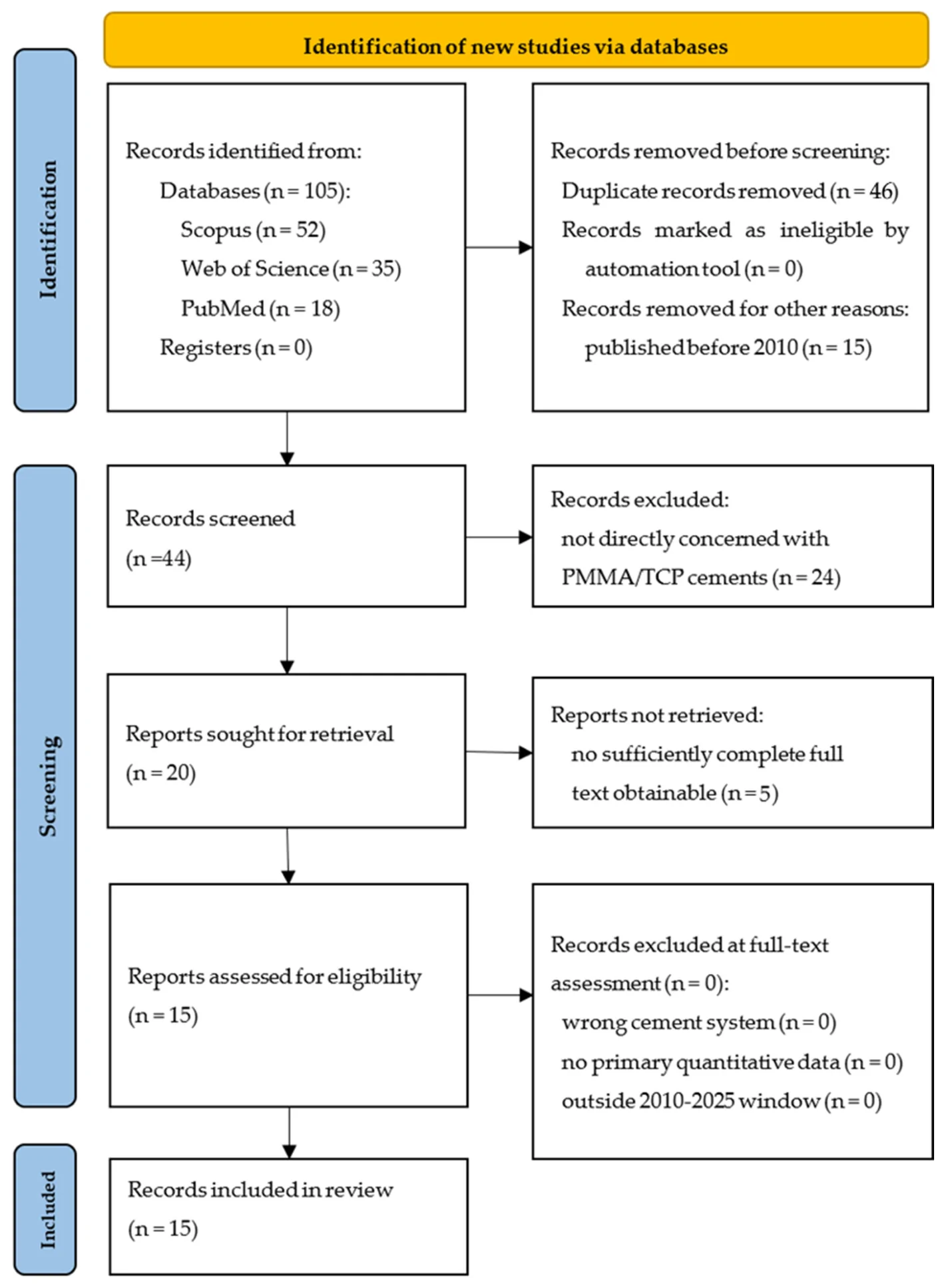
PRISMA 2020 flow diagram of the identification, screening, retrieval and inclusion of records. Adapted from the PRISMA 2020 flow diagram for new systematic reviews that included searches of databases and registers only, reproduced under the terms of the Creative Commons Attribution (CC BY 4.0) licence.

The selection process is summarised quantitatively in the PRISMA flow diagram (Figure 3). The initial searches returned 105 records (Scopus, *n* = 52; Web of Science, *n* = 35; PubMed, *n* = 18).

Before screening, 61 records were removed: 46 duplicates identified across the databases and a further 15 records published before 2010. The remaining 44 records were screened at the title/abstract level, of which 24 were excluded because—despite matching the search terms—they did not directly address PMMA/TCP cements. These records are not cited individually, since none of them met the inclusion criteria on any reading. This left 20 reports sought for retrieval, of which six could not be obtained (predominantly because of subscription restrictions). The five reports that could not be obtained are cited individually [48,49,50,51,52], each having appeared, on the basis of its title and abstract, to satisfy the inclusion criteria; their eligibility could not be confirmed, and no data could be extracted from them.

The resulting 15 reports were assessed for eligibility at the full-text level, and all 15 satisfied the inclusion criteria, yielding the final corpus of 15 studies analysed in this review on the impact of TCP content in PMMA cements on the modification of their basic functional properties: mechanical strength, osteointegration-related properties and additional performance characteristics.

Selective non-reporting of measured outcomes was identified in the majority of the included studies: numerical values, measures of dispersion, or both were absent for at least one outcome named in the methods of the individual reports, as recorded in Table 1 and Table 2. The mechanical synthesis was most affected; several studies described the direction of change in compressive or flexural behaviour without reporting the underlying values. Outcomes from reports assessed as potentially eligible that could not be obtained in full text are unknown, and their absence may bias any of the three syntheses in an undetermined direction. Because no quantitative synthesis was undertaken, the effect of these missing results cannot be estimated, and the syntheses should be read as descriptions of the available reported evidence rather than of all evidence generated.

The characteristics of the fifteen included studies are summarised in Table 1.

### 3.2. TCP—Tricalcium Phosphate

An analysis of the rapidly developing research on the modification of PMMA bone cements using TCP to enhance biomedical interactions and strength parameters should begin with an introduction to the additive material itself: tricalcium phosphate (Ca_3_(PO_4_)_2_). TCP is an inorganic compound composed of calcium (Ca^2+^) and phosphate (PO_4_^3−^) ions in a molar ratio of 3:2. Chemically, it belongs to the group of calcium orthophosphates, which are the basic mineral component of bone [53]. It occurs in several polymorphic forms, the most important of which are α-TCP and β-TCP, which differ in their crystal structure and physicochemical properties [54].

α-TCP has a monoclinic crystallographic structure, space group P2_1_/a. Its monoclinic unit cell contains 24 formula units, with 72 calcium atoms distributed over 18 crystallographically independent cation sites and a correspondingly high number of phosphorus sites. The volume of the unit cell is approximately 4200 Å^3^, and the density of the material is approximately 2.86 g/cm^3^. β-TCP crystallises in a different, rhombohedral (trigonal) system, space group R3c. Its structure contains five crystallographically distinct calcium sites and three distinct phosphorus sites, which makes it less complex than the α-TCP structure. The unit cell is smaller (approximately 3527 Å^3^) and, crucially, more densely packed: the theoretical density of β-TCP (≈3.07 g/cm^3^) is about 7% higher than that of α-TCP (≈2.86 g/cm^3^). The looser atomic packing of the metastable α phase is directly responsible for its higher specific surface energy, greater solubility and faster hydrolysis, whereas the denser, thermodynamically stable β lattice underlies its slower, more controlled resorption [55]. It should be noted that the reverse β→α transformation at approximately 1125 °C is accompanied by an approximately 7% volume expansion, which generates internal stresses and can induce microcracking in sintered TCP—an effect distinct from the density difference itself and relevant to the mechanical integrity of the ceramic phase. Obtaining the desired structure, i.e., the transformation of α-TCP into β-TCP, is possible, occurs at a temperature of approximately 1125 °C, and is reversible [56] (Table 3).

**Table 1:** Key papers focusing on PMMA modification—formulation, study design, comparator, and methodological context of the studies included in the review.

Authors	PMMA Base/System	TCP Phase/Additives	TCP Content	TCP/Pore Size	Porosity/Degradation	Mechanical Test Methods	Study Type	Biological Model/Duration	Comparator/Control	Sample Size/Key Reporting Limitation
Goto K, Kawanabe K, Kowalski R et al. [57]	PMMA/methyl methacrylate–methacrylate copolymer	β-TCP + inert dicalcium phosphate base	Powder fraction 61.8 wt%; β-TCP content 49.2 wt% of powder	β-TCP particles fragmented at bone interface; no explicit grain size	Medial tibial cortex under frame shows cortical porosis/thinning; limited degradation of TCP at interface up to 16 weeks	Detaching test (bone–cement tensile/bond strength); histology, SEM, EDX	In vivo	Japanese white rabbits, tibial cortex, 4–16 weeks	G2B1 composite cement versus CMW1 PMMA cement	*n* = 10; no complete ISO-standard bulk mechanical or fatigue assessment
Lan CW, Niu GCC, Chang SJ, Yao CH, Kuo SM [58]	Commercial PMMA cement	Chitosan–TCP microspheres (β-TCP inside chitosan gel)	TCP fraction NR; microspheres as discrete filler	Microspheres 200–800 μm; pores ~200–250 μm in chitosan sponge/tooth plug	Modified PMMA becomes rough and porous after gradual degradation of chitosan–TCP microspheres	Compression and bending noted qualitatively; T_max_, setting time measured	In vitro + in vivo (cranial/condyle defects)	Rabbits (cranial and condyle defects) in previous Kuo/Lin/Chang studies cited in review	Commercial PMMA versus PMMA containing chitosan–TCP microspheres	NR; mechanical outcomes incompletely quantified; combined chitosan–TCP additive; limited protocol detail
Lye KW, Tideman H, Wolke JCG et al. [59]	Porous PMMA (pPMMA)	pPMMA + β-TCP	~50% porosity	Pores ≈600 μm	TCP fully resorbed by 12–15 weeks	Polymerisation temperature only	In vivo	NZW rabbits, mandible, 4–15 weeks	Porous PMMA versus porous PMMA–β-TCP; irradiated and non-irradiated groups	NR; no empty-defect control; unloaded model; no complete compression/flexural dataset
Dall’Oca C, Maluta T, Cavani F et al. [60]	NP PMMA cement (Mendec)	β-TCP (porous P cement)	30 wt% β-TCP	Macropores 100–250 μm; micropores 10–15 μm	Bone ingrowth 250–400 μm; superficial only	Compression, bending, bending modulus	In vivo	NZW rabbits, femur, 8 weeks	Non-porous PMMA versus porous PMMA–TCP	*n* = 8 (16 femurs/2 cements); no fatigue testing; below ISO 5833 compression and flexural-modulus thresholds
Cojocaru I, Prodan D, Popescu V, Moldovan M [61]	PMMA powders	TCP + chitosan or HA + chitosan	~40 wt% CH + TCP or CH + HA	Fine TCP/HA; porous microstructure	NR (porous 3D network)	Curing peak temperature, setting time	In vitro	Materials only	PMMA composites containing TCP–chitosan or HA–chitosan phases	NR; morphology/handling focus; no full quantitative mechanical or biological assessment
Dall’Oca C, Maluta T, Micheloni GM et al. [62]	C PMMA (Mendec Spine)	β-TCP powder (P), powder + granules (PG)	P: 26.1% TCP; PG: powder + 8.3% granules	Granules 100–300 μm; powder 53 μm	PG: progressive filling, osteoid and osteons by 12 months	Histology + ESEM/EDS; no full ISO mechanics	In vivo	NZW rabbits, femur, 1–12 months	PMMA, PMMA with TCP powder, and PMMA with TCP powder–granule formulations	NR (24 femurs/6 periods/3 cements); semi-quantitative histology; no complete standardised mechanical or fatigue dataset
Zhang X, Kang T, Liang P, Tang Y, Quan C [63]	PMMA	Biphasic CaP (HA + β-TCP)	Increasing β-TCP fraction in BCP	Granules ~100–300 μm	Higher β-TCP → more porosity after PBS	Compression, nanoindentation	In vivo	Rabbit bone defects	PMMA versus BCP–PMMA formulations with varying HA:β-TCP composition	*n* = 3/*n* = 5; BCP composition prevents isolation of a β-TCP-specific effect
Gao S, Lv Y, Yuan L et al. [64]	PMMA	Nano-β-TCP + HEMA	50 wt% TCP; HEMA 0–20%	TCP ~100 nm	BV/TV↑ in THP vs. PMMA at 12 weeks	Compression, flexural, T_max_, setting; pull-out	In vitro + in vivo	MC3T3-E1; NZW rabbits, femur, 4–12 weeks	PMMA; PMMA–β-TCP without HEMA; PMMA–β-TCP–HEMA; unaugmented screw control for pull-out testing	Setting *n* = 3; compression/flexural *n* = 5; bone-ingrowth model *n* = 3 rabbits/group; pull-out model *n* = 12 rabbits/group initially, *n* = 6 legs/group/time point; β-TCP and HEMA effects partly confounded; no fatigue/ageing test
Ilhan E, Karahaliloglu Z, Kilicay E et al. [41]	PMMA (Oliga1); TCP (Neocement)	PS-Agsbox AgNPs	1.25–3.75 wt% NPs	NPs ~193 nm; TCP cement more porous	PMMA porosity 12→71% (0→15% NPs)	Compression, water uptake, Ag release	In vitro	MC3T3-E1; *E. coli*, *S. aureus*	PMMA and α-TCP cements with 0, 1.25, 2.5, or 3.75 wt% PS–Agsbox nanoparticles	*n* = 3; AgNPs confound antibacterial and biological effects
Fang CH, Lin YW, Sun JS, Lin FH [65]	PMMA (Pure P)	TCP + chitosan (65/35)	PMMA:TCP/CH 3:1→1:3	Pores ~200 μm after degradation (P1TC3)	P1TC3: 12.8% mass loss/60 days; others 2.6–9.1%	Compression, modulus, shear, T_max_, setting	In vitro + in vivo	L929; SD rats calvaria, 4 weeks	Pure PMMA versus PMMA–TCP–chitosan formulations (P3TC1–P1TC3)	Mechanical tests *n* = 12; animal group size NR; TCP and chitosan varied together; short 4-week in vivo follow-up
Russo T, De Santis R, Gloria A et al. [66]	PMMA cranioplasty cement	Bioactive glass + Cu-β-TCP	NR	Granules 90–355 μm	Macroporosity (SEM); NR numeric	Compression, bending, ISO 5833	In vivo	Rabbit skull, 6 weeks	Reference PMMA versus PMMA containing Cu-doped TCP and bioactive glass	NR (*n* ≥ 5—ASTM D790); rabbit skull model, 6 weeks; multi-component formulation prevents isolation of TCP effect; short follow-up
Wekwejt M, Michalska-Sionkowska M, Bartmański M et al. [67]	PMMA (Cemex)	TCP + biodegradable fillers	TCP 2.5–10 wt%	TCP ≈4 μm; pores up to ~10% vol.	Porosity increases with filler; mass loss NR	Compression, microhardness, nanoindentation	In vitro	DPSC; *S. aureus* (with AgNP)	Unmodified PMMA; PMMA with TCP or other biodegradable modifiers; AgNP/gentamicin-containing controls	Compression/hardness *n* = 5; PBS exposure *n* = 3; nanoindentation *n* = 10; in vitro only; multiple modifiers and antimicrobial additives preclude TCP-specific attribution
Karahaliloglu Z, Kilicay E [68]	PMMA (Cemex/Oliga)	TCP (KyphOs) + PC-SeNPs	SeNPs 0.625–3.75 wt%	SeNPs ~103 nm	TCP cement slower, prolonged Se release	Compression, deformation of wet samples	In vitro	MC3T3-E1, SaOS-2; bacteria	PMMA and TCP-based cements with varying SeNP content	NR; SeNP loading and cement composition confound TCP-specific attribution
Maluta T, Lavagnolo U, Segalla L et al. [69]	PMMA-based Calcemex	CaP/TCP-type fillers	NR	NR	NR	Compression, bending, interface tests	In vivo	Large animal models	Calcemex formulations and reference material, as reported	*n* = 10; formulation composition and some protocol details incompletely reported; no fatigue analysis
Karpiński R, Szabelski J, Krakowski P et al. [33]	Heraeus Palamed PMMA	α-TCP, β-TCP powders	0–10 wt%	TCP grains 15–30 μm	NR (microcracks by SEM)	Compression, Young’s modulus, Shore D hardness	In vitro	Materials only	Unmodified PMMA; α-/β-TCP concentration series	*n* = 7–8; in vitro only; no ageing, fatigue, or biological validation

**Table 2:** Normalised key mechanical metrics—safe TCP ranges and strength penalties (NR = not reported in the source study).

Source	Key Quantitative Outcomes (Incl. Mechanics)
Goto K, Kawanabe K, Kowalski R et al. [57]	Bone bonding strength (kgf/cm^2^): G2B1 43.9 ± 2.0 (4 w), 86.7 ± 2.6 (8 w), 128.7 ± 2.9 (12 w), 64.9 ± 2.6 (16 w) vs. PMMA CMW1 30.8 ± 1.0, 51.5 ± 1.4, 64.9 ± 1.9, 64.9 ± 2.6 (1 kgf/cm^2^ = 0.0981 MPa); G2B1 significantly higher at all time points. Interface: G2B1 contacts bone directly without soft tissue layer; CMW1 always has a fibrous layer between cement and bone. Morphology: New bone forms on G2B1 surface and cortex by 4 weeks; gap fills with bone by 8 weeks; bone intrusions into cement surface at 8–12 weeks; cortical porosity beneath frame more marked with G2B1 due to stress shielding. Degradation: Fragmented β-TCP particles seen at margin of newly formed bone; degradation slower than in medullary canal models.
Lan CW, Niu GCC, Chang SJ, Yao CH, Kuo SM [58]	Polymerisation: Curing peak temperature significantly decreased vs. pure PMMA; setting time increased from ≈3.5 min (PMMA) to ≈9 min with chitosan–TCP microspheres. Mechanical: Ultimate compressive and bending strength reduced compared to pure PMMA, limiting use as load-bearing cement. Biology: Composites degrade gradually, create rough/porous spaces beneficial for cell adhesion and bone ingrowth; chitosan–TCP PMMA implants show bone filling of cranial/condyle defects in rabbits.
Lye KW, Tideman H, Wolke JCG, et al. [59]	Mechanical: No ISO σ_c_; polymerisation T_max_ reduced vs. conventional PMMA (NR exact value). Biology: ~70% defect bridging by bone; thin fibrous interface; no difference between pPMMA vs. ptcpPMMA in bone fill or nerve safety.
Dall’Oca C, Maluta T, Cavani F et al. [60]	Compressive strength: NP ≈ 100 MPa vs. P ≈ 50 MPa. Bending: NP ≈ 50 MPa vs. P ≈ 22 MPa; modulus: NP ≈ 3100 MPa vs. P ≈ 900 MPa. Osteointegration: Affinity index, 80.0 ± 4.3% (P) vs. 21.2 ± 1.8% (NP); bone and vessels into pores up to 400 μm.
Cojocaru I, Prodan D, Popescu V, Moldovan M [61]	Curing peak temperature: Significantly reduced vs. plain PMMA (exact °C not tabulated); setting time: Increases from 4 to 7 min. Mechanical: No explicit σ_c_ or bending values; qualitative note that high filler may risk non-homogeneous distribution and aggregation.
Dall’Oca C, Maluta T, Micheloni GM et al. [62]	Osteointegration grading: C = 0 at all times (no penetration); P: 1→2 (limited organic penetration); PG: 1.2→5 (widespread osteoid/osteons inside cement). Mechanical context: Cylinders remain stable in bone for 12 months; no numeric σc reported, implying acceptability for void filler (not for high load).
Zhang X, Kang T, Liang P, Tang Y, Quan C [63]	Compressive strength: Decreases with higher CaP; typical values remain >50 MPa (void filler range). Biology: Higher β-TCP increases ALP, mineral deposition and new bone formation around cement.
Gao S, Lv Y, Yuan L et al. [64]	Compressive strength: PMMA 57.6 ± 4.0 MPa; THP5000 (50% TCP, 0% HEMA) 72.8 ± 2.6 MPa; THP5010 (10% HEMA) further increased (≈75–80 MPa, exact mean not separately printed but higher than THP5000). Flexural strength: PMMA 45.0 ± 5.3 MPa; THP5000 33.0 ± 4.1; with HEMA up to 10–15%, flexural strength climbs back to ≈45 MPa (THP5015 ≈ PMMA). Pull-out strength: Blank 434.8 ± 50.2 N; PMMA 804.5 ± 109.2 N; THP5000 509.7 ± 124.9 N; THP5010 743.1 ± 136.2 N at 4 weeks, with THP groups increasing over time. Biology: BV/TV significantly higher for THP5000/5010 vs. PMMA; clear bone ingrowth into cement domains in THP5010.
Ilhan E, Karahaliloglu Z, Kilicay E et al. [41]	Compressive strength of PMMA: Increases from ~146 MPa (no NPs) to ~237 MPa at 5 wt% NPs (then plateaus/decreases slightly). Compressive strength of TCP cement: Increases from ~2.6 to ~8.3 MPa with NPs. Water uptake: Higher for TCP than PMMA; porosity rises strongly with NPs in PMMA. Biology: Enhanced antibacterial effect (TCP + Ag > PMMA + Ag) without loss of cell viability.
Fang CH, Lin YW, Sun JS, Lin FH [65]	Compressive stress: Pure P 127.4 ± 16.8 MPa; P3TC1 109.0 ± 0.6; P2TC1 107.5 ± 4.9; P1TC1 108.8 ± 3.5; P1TC2 90.3 ± 6.7; P1TC3 72.1 ± 6.2 MPa (*n* = 12). Modulus: Decreases from ≈1278 MPa (Pure P) to ≈1042 MPa (P1TC2) and ≈1019 MPa (P1TC3). Shear force to metal: Pure P 1266 N; P1TC2 ~1037 N; P1TC3 ~936 N. Biology: More pores and better bone ingrowth with higher TCP/CH; significant narrowing of calvarial defect for P1TC2/P1TC3 in 4 weeks.
Russo T, De Santis R, Gloria A et al. [66]	Mechanical: Compressive and flexural strength within ISO 5833 limits, comparable to reference PMMA cranioplasty cement (exact MPa not fully tabulated). Biology: Cu-TCP + bioactive glass significantly enhance bone ingrowth and direct contact vs. PMMA alone.
Wekwejt M, Michalska-Sionkowska M, Bartmański M et al. [67]	Compressive strength: Moderate decrease with higher filler; 5 wt% composite (incl. TCP) highlighted as keeping σc and hardness within acceptable range (exact MPa varies by formulation, typically >60 MPa). Microhardness: Slight reduction with porosity; nanoindentation modulus decreases modestly. Biology: 5 wt% gives best balance of porosity, mechanics and cytocompatibility.
Karahaliloglu Z, Kilicay E [68]	Compressive strength: Decreases with porosity and SeNP loading, but remains within clinically usable range (exact MPa by group not fully tabulated). Wet deformation: Higher in more porous SeNP-loaded PMMA/TCP, indicating lower stiffness. Biology: SeNPs improve antibacterial performance and selectively induce apoptosis in SaOS-2, while TCP + Se supports higher ALP and mineralisation.
Maluta Maluta T, Lavagnolo U, Segalla L et al. [69]	Mechanical: Compressive and bending strength adequate for spinal/orthopaedic cement, comparable to standard PMMA cements (exact MPa not fully tabulated). Biology: Good osteointegration and biomechanical anchorage at bone–cement interface.
Karpiński R, Szabelski J, Krakowski P et al. [33]	Compressive strength: PMMA ≈ 67 MPa; unchanged at low TCP contents. α-TCP at 3 wt% reduced CS by ~8.5% (compared to unmodified PMMA); β-TCP—no statistically significant reduction up to 10 wt%. Stiffness: ≈1400 MPa (PMMA), β-TCP up to ≈1640 MPa at 8%; α-TCP ~1200–1300 MPa Hardness: Shore D unchanged across 0–10% TCP.

**Table 3:** Systematic comparison of α-TCP and β-TCP polymorphs: from crystal structure through solubility and resorption to mechanical impact in PMMA cements.

Property	α-TCP	β-TCP
Crystal system/space group	Monoclinic, P2_1_/a	Rhombohedral (trigonal), R3c
Thermal stability	Metastable at room temperature; stable 1125–1430 °C	Stable at room temperature up to ≈1125 °C
Theoretical density	≈2.86 g/cm^3^	≈3.07 g/cm^3^ (≈7% higher)
Solubility (pKsp, 25 °C)	Higher; pKsp ≈ 25.5	Lower; pKsp ≈ 28.9
Reactivity in aqueous environment	Rapid hydrolysis to CDHA (basis of CPC cements)	Essentially no hydrolysis; persists longer
In vivo resorption mechanism/rate	Fast; block degradation visible after ≈4 weeks	Slower, cell-mediated (osteoclastic), synchronised with bone formation; only slight after 8 weeks
Effect on PMMA cement strength	Reduces mechanical strength already above ≈3 wt% [33]	Mechanically safe up to ≈10 wt% [33]
Preferred application	Fine powder for self-setting CPC cements	Load-bearing granules/blocks; osteoconductive admixture

NR indicates not reported or not reported in sufficient detail in the source publication. Sample-size data refer to the specific outcome where indicated. The comparator and limitation columns provide a structured qualitative appraisal and do not constitute a validated numerical risk-of-bias score. Although both polymorphic forms of TCP differ in structure, they have an identical chemical formula and exhibit similar biocompatibility properties. Both α-TCP and β-TCP can be used as bone regeneration materials due to their significant structural and functional similarities to the natural mineral components of human bone. Most importantly, they show significant similarity to hydroxyapatite (Ca_10_(PO_4_)_6_(OH)_2_), which is the main mineral component of human bones [70,71]. They differ primarily in their Ca/P ratio, which is 1.5 for TCP and 1.67 for hydroxyapatite. Furthermore, TCP can transform into hydroxyapatite in a physiological environment, mimicking the natural mineralisation processes that occur in bone tissue [70]. TCP is similar in composition and function to natural calcium phosphates found in bones (i.e., hydroxyapatite and carbonate apatite, among others). β-TCP elicits a similar phagocytic cellular response via osteoclast-like cells as natural bone tissue [72]. Both TCP polymorphisms are biodegradable and can be gradually replaced by natural bone tissue in the body [56,70,73]. The remaining α-TCP particles surrounded by newly formed bone material are gradually absorbed by osteoclast-like cells [56].

Despite having identical chemical compositions, the structural differences described above, α-TCP and β-TCP, exhibit significant physicochemical differences. β-TCP is stable at room temperature, while α-TCP is metastable and can be obtained by rapid cooling from high temperatures. The higher solubility of α-TCP is governed primarily by its higher lattice energy and thermodynamic metastability rather than by density alone: the solubility product of α-TCP (pKsp ≈ 25.5) is markedly higher than that of β-TCP (pKsp ≈ 28.9). Consequently, in an aqueous or physiological environment, α-TCP is far more reactive and readily hydrolyses to calcium-deficient hydroxyapatite (CDHA)—a reaction that is the operating principle of self-setting calcium-phosphate cements but is essentially absent for β-TCP.

This chemical reactivity translates directly into resorption behaviour, as demonstrated by histological and histomorphometric studies. When analysing TCP blocks in the form of solid, three-dimensional structures as models for evaluating biological properties such as biodegradation, bioresorption or the ability to support bone tissue regeneration, it was shown that α-TCP degrades much faster than β-TCP. The degradation of α-TCP blocks was noticeable after only 4 weeks, while β-TCP blocks showed only a slight degree of degradation even after 8 weeks. Importantly, in vivo β-TCP is not simply dissolved but undergoes cell-mediated (osteoclastic) resorption at a rate that tends to match new-bone formation, which is a key reason for its preference in load-supporting bioceramics [56].

The mechanism of action of TCP-modified PMMA cement can be described as a sequence of coupled physicochemical and biological processes. The setting of the acrylic matrix itself is a cold, free-radical chain polymerisation of methyl methacrylate (MMA). It is initiated by the redox decomposition of benzoyl peroxide (BPO) in the presence of a tertiary aromatic amine activator (typically N,N-dimethyl-p-toluidine, DMPT), which generates benzoyloxy radicals already at body/room temperature: BPO + amine → 2 R^•^. These radicals add to the C=C double bond of MMA (initiation), triggering rapid propagation (R^•^ + n MMA → R-(MMA)_n_^•^) and finally termination by combination or disproportionation, producing solid poly(methyl methacrylate) that entraps the pre-polymerised PMMA beads and the ceramic filler. Because propagation is strongly exothermic (the enthalpy of MMA polymerisation is approximately −57 kJ/mol), the reaction releases the heat responsible for the polymerisation exotherm; the amount and rate of radical generation, governed by the BPO/amine concentrations, directly control the setting time, the degree of double-bond conversion and the peak temperature [74]. In this scheme, the dispersed TCP particles act primarily as an inert-to-reactive filler that dilutes the exothermic monomer fraction and provides a heat sink, which contributes to the lower polymerisation peak.

Once the composite is in contact with body fluids, the bioactive mechanism proper is driven by the ceramic phase. TCP particles exposed at the cement surface and within the interconnected porosity partially dissolve, releasing calcium and orthophosphate ions into the surrounding medium according to the equilibrium Ca_3_(PO_4_)_2_ ⇌ 3 Ca^2+^ + 2 PO_4_^3−^. This dissolution is markedly faster for the metastable α-TCP than for the denser β-TCP, in line with their solubility products (pKsp ≈ 25.5 vs. 28.9). The locally elevated Ca^2+^ and PO_4_^3−^ activities raise the ionic supersaturation of the interfacial fluid with respect to apatite, which first precipitates as a transient amorphous calcium phosphate (ACP) phase and subsequently matures into a carbonated, calcium-deficient hydroxyapatite (CDHA) layer [75]. The nucleation and growth of this bone-like apatite layer follows the classical Kokubo mechanism: surface functional groups and released ions create apatite nuclei, which then grow spontaneously by consuming Ca^2+^ and PO_4_^3−^ from the supersaturated fluid: 10 Ca^2+^ + 6 PO_4_^3−^ + 2 OH^−^ → Ca_10_(PO_4_)_6_(OH)_2_ [76]. It is precisely this in situ apatite layer that establishes a direct chemical bond between the cement and the host bone—the physicochemical basis of the bioactivity (osteoconduction and osseointegration).

Over longer time scales, the TCP phase is not merely dissolved but is actively remodelled together with the surrounding tissue. The apatite/TCP interface is recognised by bone cells: osteoclast-like cells adhere to the ceramic, acidify the local microenvironment (via a proton pump and carbonic-anhydrase-mediated H^+^ secretion) and enzymatically resorb the mineral, further releasing calcium and phosphate ions. This osteoclastic resorption is spatially and temporally coupled to osteoblastic new-bone deposition, so that the resorbed β-TCP is gradually replaced by newly formed bone matrix at a rate that, for β-TCP, tends to match the pace of bone formation [77]. The released ions, in turn, feed back into the local mineralisation pool, sustaining apatite formation and creating a self-reinforcing loop between filler resorption and bone remodelling. Taken together, these steps—radical polymerisation of the PMMA matrix, dissolution of TCP with Ca^2+^/PO_4_^3−^ release, supersaturation-driven precipitation of an apatite layer, and cell-mediated resorption coupled to bone remodelling—constitute the integrated mechanism of action that links the composition and microstructure of PMMA/TCP cements to their mechanical, biological and functional performance.

The steps above explain how the composite bonds to bone, but its clinical performance also depends on a second, internal interface—that between the TCP filler and the PMMA matrix—which controls how mechanical load is transferred and how fracture proceeds. Unlike the pre-polymerised PMMA beads, which are chemically wetted and partially dissolved by the MMA monomer during setting, the hydrophilic ceramic surface is intrinsically incompatible with the hydrophobic, non-polar PMMA network; there is no covalent continuity across the TCP–polymer boundary, and adhesion relies on comparatively weak mechanical interlocking and secondary interactions. This chemical mismatch is the molecular origin of the poor filler–matrix compatibility discussed later to explain the drop in flexural strength of TCP/HEMA/PMMA cements and its restoration once HEMA is added as a compatibiliser [64], and it is consistent with the increase in surface hydrophobicity (contact angle rising from ≈65° to ≈123°) recorded when TCP is incorporated [67]. Because stress is transmitted from the compliant matrix to the stiff particles only across this interface, its quality sets an upper bound on how efficiently the ceramic can reinforce—rather than merely fill—the cement; the same reasoning underlies the use of coupling agents, which chemically graft filler and matrix and are known to raise the transverse strength of particulate/fibre-reinforced PMMA by improving precisely this load transfer [78].

At the same time, the ceramic phase and the porosity it generates govern how the composite fails once a defect is present. Rigid TCP particles are stiffness- and strength-mismatched with the surrounding PMMA, so under load the interface acts as a stress concentrator: where bonding is strong, an advancing crack is forced to deflect around or bridge the particles, dissipating energy and raising the effective fracture toughness, whereas at weakly bonded or agglomerated particles the crack initiates by interfacial debonding and then coalesces through the matrix, lowering toughness and fatigue life. This particle–crack interaction has been imaged directly in radiopacifier-filled PMMA cements, where cracks preferentially nucleate at filler agglomerates [79] and either circumvent or cut through the dispersed beads depending on interfacial cohesion [80]. Superimposed on this is the deliberately introduced or dissolution-generated porosity: each pore is a pre-existing flaw that concentrates stress and shortens the crack-initiation stage, and increasing porosity has been shown to reduce the fracture toughness and accelerate fatigue crack propagation of acrylic bone cement [81]. The macroscopic trends are the direct expression of this micro-mechanism: the steep fall in compressive strength and flexural modulus of porous PMMA/β-TCP (to ≈50 MPa and ≈900 MPa) [60], and the progressive strength and stiffness loss with rising TCP/chitosan content [65], both follow from the combined effect of weak filler–matrix load transfer and the added flaw population. Conversely, the fact that β-TCP can be tolerated up to ≈10 wt% without a statistically significant strength penalty, while α-TCP above ≈3 wt% reduces compressive strength [33], reflects the slower dissolution of the denser β lattice, which limits both interfacial degradation and the rate at which new porosity—and hence new crack-initiation sites—is created.

### 3.3. Modification of Biological Activity and Osteogenesis

The introduction of tricalcium phosphate (TCP), especially its β variant in the form of BCP (β-TCP/HA) biphasic materials, significantly increases the bioactivity of PMMA-based acrylic cements. The presence of a resorbable mineral phase stimulates the differentiation of mesenchymal cells towards osteogenesis and supports mineralisation and the exchange of calcium and phosphate ions. Unlike bioinert PMMA, BCP/PMMA cements exhibit osteoconductive properties and gradual integration with bone tissue, allowing the material to be remodelled towards newly formed bone. These properties make modified PMMA/TCP composites promising materials for bone defect reconstruction and for fixing implants requiring long-term biological stability.

Interpretation of the evidence requires a clear distinction between formulations in which TCP is the principal variable and multi-component composites in which TCP is combined with additional modifiers. In the latter systems, measured changes in mechanical behaviour, porosity, degradation, cellular response, polymerisation kinetics, or antimicrobial activity cannot be attributed to TCP alone unless the study design includes appropriate composition-matched controls. Hydroxyapatite within biphasic calcium phosphates may modify dissolution and osteogenic behaviour. HEMA may alter filler–matrix compatibility. Chitosan may influence porosity, degradation and setting behaviour. AgNPs, SeNPs or Cu-containing phases may determine antimicrobial activity and may also affect cytocompatibility and mechanical properties. Accordingly, findings from such systems are interpreted in this review as properties of the complete formulation, whereas direct TCP-specific conclusions are reserved for studies comparing PMMA with otherwise equivalent PMMA-TCP compositions.

Studies have shown that porous acrylic cement composed of PMMA and β-TCP exhibits excellent biocompatibility, and a high degree of osseointegration has been confirmed by the growth of newly formed bone tissue inside the cement sample. No signs of local or systemic toxicity were observed. Cements containing TCP have a higher affinity index compared to standard PMMA cements. For porous PMMA/β-TCP cements, this index averaged 80.01 (±4.3), while for non-porous PMMA cements it was only 21.24 (±1.8). These differences were statistically significant (*p* < 0.01) and indicate better integration of the material with bone tissue. One of the most important features of cements with TCP additives is their porous structure. Porous PMMA/β-TCP cements are characterised by the presence of micropores with a diameter of 10–15 μm and macropores with a size of 100–250 μm. This structure resembles the natural conditions found in cancellous bone, where bone trabeculae are spaced approximately 200–300 μm apart. Microscopic examination of the surface of porous PMMA/β-TCP cements revealed their fibrous appearance, with connected spaces and the presence of numerous micro-reliefs, cavities and pores [60].

In the BCP–PMMA system investigated by Zhang et al. [63], an increased β-TCP proportion within the HA–β-TCP phase was associated with higher osteogenic-marker expression and mineralisation. Because the total ceramic content, HA:β-TCP ratio, and resulting microstructure may all influence the response, these results support a formulation-level association rather than an isolated β-TCP effect. A higher β-TCP content (e.g., 70%) correlated with more intense alkaline phosphatase (ALP) expression and extracellular matrix mineralisation, as confirmed by Alizarin Red tests. In an in vivo model of radial bone defect in rabbits, BCP/PMMA cements induced faster bone tissue regeneration than pure PMMA and did not cause inflammatory reactions. After 26 weeks, the volume of new bone (BV/TV) in the BCP7/PMMA group was 52.58%, compared to 36.47% for PMMA. β-TCP accelerates cement resorption due to its higher solubility compared to HA. In BCP/PMMA cements, gradual material loss was observed in micro-CT, especially at higher β-TCP content (70%), which correlated with the replacement of cement by new bone. Unlike PMMA, which remains biocompatible, β-TCP cements showed dynamic exchange of calcium and phosphate ions, promoting mineralisation, and apatite crystal deposition was observed in simulated body fluid (SBF).

Histological studies revealed a key difference between TCP-containing cements and standard PMMA. G2B1, a methacrylate-based composite bone cement with a high 49.2 wt% β-TCP additive, was in direct contact with the bone without any intervening soft tissue in all study periods, whereas in the case of PMMA, there was always a layer of soft tissue separating the cement from the bone. SEM observations confirmed that fragmented β-TCP particles were present at the margins of the newly formed bone tissue after 8 weeks, indicating the osteointegrative and degradative properties of the cement. The degradation of β-TCP particles in G2B1 was visible at the margins of the newly formed bone tissue, indicating an active bone remodelling process. This aspect is particularly important for the long-term effectiveness of bone cements in cases where it is desirable to replace the implant material with natural bone tissue [57].

Karpiński et al. (2024) noted that the higher solubility of α-TCP can accelerate bone regeneration, which makes the choice of polymorph and concentration a means of tuning the biological response; the mechanical consequences of this solubility ranking are addressed later [33].

A PMMA composite containing β-TCP and HEMA showed improved bone-growth-related outcomes in a rabbit model after 12 weeks compared with unmodified PMMA. Because HEMA was introduced as a compatibilising co-monomer and altered filler–matrix interactions, the observed response should be interpreted as an effect of the β-TCP-HEMA-PMMA formulation rather than of β-TCP alone. Micro-CT showed an increased bone volume to total volume ratio (BV/TV) compared to PMMA [64].

In another study, TCP cement showed lower dentine pulp stem cell (DPSC) viability compared to unmodified PMMA, which was attributed to the release of unbound MMA monomer. However, haemolysis tests confirmed the absence of toxicity (haemolysis index < 5%). It was also noted that the addition of TCP increased the porosity of the cement, creating channels that allowed for ion diffusion (e.g., Ca^2+^), potentially supporting mineralisation processes. After 30 days in phosphate-buffered saline (PBS), larger pores (average 50–100 μm) were observed compared to pure PMMA. In formulations containing both TCP and AgNPs, antibacterial activity against *S. aureus* was observed and is attributable primarily to silver nanoparticles. The TCP-containing matrix may have modified porosity and, consequently, the release behaviour of AgNPs; however, the study did not permit the antibacterial contribution of TCP itself to be separated from that of silver (reduction in dehydrogenase activity in *S. aureus* biofilms by ~40%). The effect was comparable to cements containing gentamicin [67].

Two modified PMMA cements were evaluated in study [59]: porous PMMA (pPMMA) and porous PMMA with β-TCP additive (ptcpPMMA). Both materials demonstrated good biocompatibility and the ability to stimulate bone ingrowth into the porous structure. After 12–15 weeks of implantation in a rabbit model, 70% of the samples achieved bone defect bridging, with no significant differences between the groups. β-TCP degraded within 12–15 weeks, leading to the formation of pores and irregularities on the cement surface. This process may have promoted secondary bone ingrowth, but at the same time increased the presence of particles in the surrounding soft tissues. An inflammatory reaction was observed around the particles, which decreased as the material resorbed. In both cements, a thin layer of connective tissue was found at the bone interface, with no direct contact between the bone and PMMA. The addition of β-TCP did not improve bone adhesion to the cement. In the model with postoperative irradiation (dose 22.4 Gy), no negative effect of radiation on tissue healing was observed for either cement. Bone ingrowth indices and bone tissue maturity were comparable between the irradiated and non-irradiated groups.

In studies by Maluta et al. [69], both on liquid and pre-polymerised preparations, the formation of an even, well-organised bone capsule approximately 200 µm thick was observed around the cement, in full continuity with the trabecular bone network. Integration was more advanced in the case of cement applied in liquid form, where deeper penetration of newly formed bone tissue into the material was observed, thanks to the favourable microarchitecture and the presence of channels and resorbable TCP granules. The cement was perfectly integrated with both the cortical and cancellous bone, providing a scaffold for cell migration and the formation of new hard tissue [69].

In PMMA composites containing both TCP and chitosan, increased degradation, pore development, and changes in mechanical properties reflect the combined effects of the resorbable calcium-phosphate phase and the degradable chitosan component. The individual contribution of TCP cannot be quantified from these studies without formulation-matched controls in which TCP and chitosan are varied independently.

The mineral fraction undergoes dissolution and cellular resorption (osteoclast activity), and the resulting empty spaces are gradually replaced by newly formed bone tissue. Maluta et al. [69] described that TCP resorption was synchronised with the rate of new bone formation, without compromising the integrity of the material for at least 12 months. The final microstructure of the cement was characterised by numerous channels and spaces that provided pathways for cell and vessel migration, and the bone returning to these areas had normal histological morphology. No local inflammatory reactions, necrosis, fibrous tissue overgrowth or foreign body reactions were observed around the implanted cement. Both liquid and pre-polymerised preparations did not induce cellular infiltration, swelling or changes in the morphology of the adjacent bone. No systemic toxic symptoms were observed in the animals studied for 12 months. PMMA cements with TCP, in particular with adequate macroporosity, did not cause bone growth disorders in young test subjects, did not induce changes in the cortical bone layer, and did not lead to atrophic or degenerative changes in soft tissues. Maluta et al. reported that one year after implantation, no growth disorders or joint pathologies were found [69].

Very similar phenomena were described by Dall’Oca et al., who compared three formulations: pure PMMA (C), PMMA with TCP in powder form (P) and PMMA with TCP in powder and 100–300 µm granules (PG). The highest degree of osseointegration was achieved with PG cement—after only three months, osteoid formation, the presence of fibroblasts and bone cells were observed within the material, and in the following months, osteoid-like structures began to grow. The osteointegration process was assessed semi-quantitatively, showing a progressive increase in the degree of the phenomenon only for PG cement, where after 12 months the highest degree of material penetration by organic components (bone, osteoid, and cells) was achieved, while in PMMA and P cement, this process was severely limited to peripheral areas. The addition of TCP in powder form only improved osteoconduction to a limited extent, mainly due to the formation of small pores, while the presence of larger granules and appropriately designed macro- and micropores enabled extensive cell infiltration, vascular ingrowth and actual remodelling of the material into bone tissue.

However, the progression of bioresorption and remodelling processes over time was described in detail. The best results were achieved with PG cement, where fibrin matrix infiltration occurred as early as 2–3 months, followed by fibroblasts and osteoid, and after 6 months, osteon organisation was visible. These phenomena did not progress in P and C cements, which was attributed to the small size of the pores and the lack of effective channels allowing deep cell infiltration. Histological evaluation, based on a semi-quantitative scale of inflammation intensity (phlogosis, presence of inflammatory cells, necrosis, fibrosis, etc.), showed virtually no difference between porous and non-porous cements. Only in the first month after implantation were minimal signs of cell degeneration observed, probably related to surgical trauma, which subsided in the following months. At no time were cytotoxic reactions attributed to the cement material observed.

The implants were also confirmed to be highly identifiable in X-ray and MRI examinations throughout the experiment, although radiological differences between cements with different amounts and sizes of TCP granules were clearly visible—PG cements became less radiopaque as the mineral resorbed. In the studies by Dall’Oca et al., the introduction of TCP granules with a fraction of 100–300 µm into the matrix proved to be crucial: only in such a system was a wide clearance for cell migration achieved, which translated into the ability to completely remodel the material. For smaller particles (powder ~53 µm), this effect was marginal—the material did not undergo transformation into bone, despite the presence of a few pores.

Ultrastructural analyses performed in SEM/ESEM prove that the presence of TCP, especially in the form of granules, gives PMMA hybrid cement a characteristic structure with distinct macropores and a network of microchannels that connect to the surrounding area and, as resorption progresses, are replaced by new bone tissue. Elongated cavities form in the central regions of the cement, which are used as “bridges” for bone trabeculae, especially in the fluid variant applied in situ. By designing the size of the granules and pores, it is possible to synchronise the rate of resorption and remodelling with the natural healing processes of bone tissue. In clinical practice, this translates into better regeneration, stability and a potentially lower tendency to necrosis or foreign body reactions. In the studies discussed, these processes proceeded in full accordance with physiology, without any disturbances [60].

In study [68], incorporation of selenium nanoparticles (SeNPs) into PMMA- and TCP-based cement formulations improved antibacterial activity against *E. coli* and *S. aureus*. This antimicrobial response was attributable to SeNPs, as TCP alone did not demonstrate significant antibacterial activity in the reported experiments. TCP may have influenced the formulation’s porosity, dissolution behaviour, or selenium-release profile, but its independent antimicrobial contribution was not established. However, TCP alone did not show any significant antimicrobial activity—this effect was attributed solely to the presence of SeNPs. Cements with SeNPs (including TCP-SeNPs) increased the production of reactive oxygen species (ROS) in SaOS-2 cells (bone cancer cell line), leading to apoptosis, while showing no toxicity to healthy osteoblasts (MC3T3-E1).

Russo et al. [66] investigated a multi-component PMMA cranioplasty composite containing Cu-doped TCP and bioactive glass. The reported antibacterial activity should be ascribed primarily to copper release, while the observed biological and mechanical outcomes reflect the combined effects of the Cu-containing TCP phase, bioactive glass, ceramic-particle morphology, and the PMMA matrix. The study therefore does not isolate the effect of TCP alone. Cytotoxicity studies have shown that PMMA/Cu-TCP cements are non-toxic, as confirmed by the MTT test. Furthermore, on the surface of these materials, bone marrow mesenchymal cells (BMMSCs) were able to differentiate towards the cartilage line. An important aspect of Cu-TCP modification is to give them antibacterial properties—Cu-TCP particles are effective in limiting the growth of Pseudomonas aeruginosa bacteria (significant reduction in bacterial growth at 2.5% *w*/*w*). At a concentration of 5% by weight, Cu-TCP particles reduced the growth of *P. aeruginosa* by approximately 80% and 75%, respectively. Modification of bone cements with these particles was also effective in reducing the growth of Staphylococcus aureus, which is the most common pathogen affecting cranioplasty. However, no significant inhibition of Escherichia coli growth was observed, which may be due to the fact that *E. coli* strains can survive in copper-rich environments because they have plasmid-encoded genes that confer copper resistance [66].

Also, chitosan/β-TCP/PMMA-modified composites provide rough and porous surfaces that promote cell adhesion and growth. This porosity allows for bone ingrowth into the material, which is crucial for long-term implant stability. The addition of TCP also affects the degradability of the material. Chitosan/β-TCP/PMMA-modified composites can gradually degrade, as confirmed by in vitro degradation studies and SEM observations [58].

Analysis of the structure and morphology of PMMA/TCP composites provided interesting information. Scanning electron microscopy (SEM) images showed that the composites have a homogeneous and porous microstructure. EDX analysis confirmed the presence of C, Ca, P and O in the composites, which was consistent with the expected composition. It was also observed that PMMA molecules tend to form chains in the presence of chitosan. The inorganic phase embedded in PMMA and chitosan showed varying degrees of adhesion to PMMA spheres, with better adhesion observed in the HA/CH/PMMA composite than in the TCP/CH/PMMA composite [61].

The authors of [65] focused on the presence of TCP and chitosan in PMMA, which led to the formation of pores up to 200 µm in size, providing favourable conditions for bone cell growth and implant integration with soft and bone tissue. Increased cement bioresorption was manifested by a gradual loss of composite mass analysed after a specified incubation period in PBS. The loss over 60 days ranged from 2.6% for pure cement to 12.8% for a composition with a PMMA-to-TCP/chitosan mixture ratio of 1:3 (P1TC3). This provides space for bone tissue growth and, at the same time, does not lead to a loss of integrity and, most importantly, facilitates the integration of the cement with the host bone. This makes PMMA/TCP biocomposites potentially better for use in dynamic, metabolic environments where gradual bioresorption of the material is required. Cytotoxicity tests (the MTT method) showed that TCP and chitosan biocomposites, except for the variant with the highest additive content (P1TC3), do not exhibit significant cytotoxicity towards L929 fibroblasts, which indicates the high biocompatibility of the material. Furthermore, histopathological in vivo studies (rats) after 4 weeks showed intense osseointegration and the formation of new bone tissue in contact with the biocomposite, which was not observed in the case of pure PMMA [65].

In summary, β-TCP-modified PMMA cements represent an effective compromise between mechanical stability and bioactivity. An increased proportion of β-TCP correlates with higher expression of osteogenesis markers, mineralisation and the formation of new bone tissue in vivo. TCP cements form porous structures that allow for cell migration, vascular ingrowth and bone remodelling. Porous PMMA/TCP/chitosan hybrids exhibit high biocompatibility and promote bone regeneration while maintaining mechanical stability and limited tissue reactivity. Histological studies confirm direct contact with bone without an intermediate layer of soft tissue, which distinguishes them from classic PMMA. It has also been shown that the appropriate macroporosity and size of TCP granules (approx. 100–300 µm) determine the effectiveness of osteoconduction and the synchronisation of the rate of resorption with bone remodelling [82]. Antibacterial activity has been reported for multi-component PMMA composites containing TCP together with antimicrobial phases such as AgNPs, SeNPs, or Cu-containing particles. In these formulations, the antibacterial effect should be attributed primarily to the metallic or metal-containing additive; the independent contribution of TCP, if any, was not isolated experimentally [83]. Thus, TCP-modified cements enable dynamic processes of osseointegration and gradual bioresorption of the material, making them promising solutions in modern orthopaedics and tissue engineering.

A critical reading of the studies compiled above must, however, weigh them according to the strength of the underlying evidence, because the biological claims summarised here rest on very different levels of proof. At the lowest tier are in vitro cell-culture assays—rBMSC adhesion, proliferation, ALP expression and Alizarin Red mineralisation [63]; DPSC and SaOS-2/MC3T3-E1 viability and haemolysis testing [67]; and MTT cytotoxicity of chitosan/β-TCP systems [65]—which establish biocompatibility and osteogenic potential but cannot reproduce load transfer, vascularisation or immune complexity. Above these sit short-term small-animal studies, predominantly rabbit defect or implantation models running 8–26 weeks [59,63,64], which add genuine osseointegration, bone ingrowth and early-resorption data. Longer-term and larger models provide the most clinically relevant evidence: the 12-month follow-up of Maluta et al. [69] and the staged 3–12-month osteointegration series of Dall’Oca et al. [60] demonstrate sustained remodelling and resorption-formation coupling, while ex vivo biomechanical testing [84] probes construct behaviour under load. Crucially, the entire body of biological evidence for TCP-modified PMMA remains preclinical: no controlled clinical trial or long-term patient follow-up was identified, so translation to human use is still an extrapolation from animal and bench data. This gradient—from abundant in vitro data, through short-term animal studies, to a scarcity of long-term and an absence of clinical evidence—mirrors the well-recognised bench-to-clinic translation gap for bone biomaterials [85] and should temper the interpretation of the favourable outcomes reported above.

Four biological dimensions in particular deserve closer scrutiny than the individual studies afford in isolation:

- The immune and inflammatory response: Although most reports describe the cements as non-cytotoxic and free of foreign-body reaction over the observation window [69,86], TCP is not immunologically inert. Resorption liberates micron- and submicron-scale ceramic particles, and study [59] explicitly recorded a transient inflammatory reaction around β-TCP particles that migrated into the surrounding soft tissue and subsided only as the material resorbed. This is consistent with in vitro evidence that calcium-phosphate particles actively modulate monocyte and macrophage activation and cytokine secretion in a phase- and size-dependent manner [87,88], implying that particle size and resorption rate—not merely bulk composition—govern the local immune outcome.
- Degradation products: The dissolving TCP releases Ca^2+^ and PO_4_^3−^ together with residual unreacted MMA monomer, the latter being the most likely cause of the reduced DPSC viability noted in [67]; the biological response therefore reflects the combined action of bioactive ionic products and potentially cytotoxic organic leachables.
- Long-term ion release: The reviewed data document ion diffusion and mass loss over 30–60 days in PBS [65,67] and resorption over 8–52 weeks in vivo [59,60,63,69], but the sustained, low-level release of Ca^2+^/PO_4_^3−^ (and of co-additives such as Ag, Se or Cu ions [66,67,68]) beyond one year and its systemic clearance—remains essentially uncharacterised.
- The most consequential for device performance, the resorption of the TCP phase, is inherently a mass-removal process: each resorbed particle leaves a void that, unless promptly filled by new bone, becomes a stress concentrator and a site of crack initiation.

The favourable cases reported here all depended on resorption being temporally synchronised with new-bone formation [60,69]; where that synchrony fails, for instance with fast-dissolving α-TCP or excessive filler loading, net porosity rises faster than bone can compensate, which is the biological origin of the progressive loss of mechanical support.

The evidence that TCP incorporation enhances bone contact, ingrowth and osteoconductive potential is consistent in direction across independent in vivo studies and may be regarded as of moderate certainty, but it was obtained predominantly in multi-component formulations and in short-term small-animal models, so that neither the contribution of TCP alone nor the magnitude of the effect can be established with confidence. The attribution of antibacterial activity to the silver-, selenium- and copper-containing phases rather than to TCP is of moderate certainty, resting on studies in which these phases were the experimental variable.

### 3.4. Mechanical Properties After TCP Admixture

The analysed studies demonstrate the significant impact of TCP on the strength parameters of PMMA-based bone cements. Given the above-described beneficial biointegrative effect, it would be sufficient if the admixture did not impair strength, especially in the long term. Depending on the type, concentration, and crystalline form of the admixture, the following studies analysed the compressive strength, flexural strength, and elastic modulus of the cement, as well as interactions with other admixtures, such as hydroxyapatite in the form of a biphasic phase (BCP) or chitosan. The selection of the optimal concentration and form of TCP therefore allows a compromise to be achieved between the mechanics and bioresorbability of the composite.

The crystalline form is itself a primary determinant of mechanical outcome. Because α-TCP dissolves and hydrolyses rapidly, even modest additions generate early porosity and local mass loss that degrade the load-bearing matrix; this is consistent with reports by Karpiński et al. [33] that α-TCP at 3 wt% reduced quasi-static compressive strength by approximately 8.5% relative to unmodified PMMA, whereas β-TCP did not produce a statistically significant reduction at contents up to 10 wt%—a threshold that, as detailed below, is itself conditional on porosity and loading mode. In other words, the same solubility ranking (α > β) that governs bioactivity and resorption also governs the rate at which the ceramic phase compromises mechanical integrity—bioactivity and mechanical durability are coupled through the dissolution behaviour of the polymorph; however, the experimental data establish an association only for the specific solid PMMA formulation and short-term testing conditions examined.

In PMMA composites containing biphasic calcium phosphate (BCP), the mechanical response depends on the total ceramic loading and the HA:β-TCP ratio. In the cited formulation, a BCP-containing composite met the ISO 5833 compressive-strength requirement; however, this result represents the behaviour of the complete HA–β-TCP composite and cannot be attributed to β-TCP alone. The composition containing 40% BCP (including 70% β-TCP) achieved values above 70 MPa, meeting the requirements of ISO 5833. A higher β-TCP content slightly reduced the strength, which was attributed to the brittleness of β-TCP particles, but the values met the reported mechanical threshold under the specific test conditions [63].

In study [64], TCP/hydroxyethyl methacrylate (HEMA)/PMMA (THP) composite cements with 50% β-TCP increased compressive strength from 57.6 MPa for pure PMMA cement to 72.8 MPa. However, flexural strength decreased (33.0 MPa vs. 45.0 MPa for PMMA), which was attributed to poor compatibility between hydrophilic TCP and hydrophobic PMMA. Within the β-TCP-HEMA-PMMA series, adding HEMA to the β-TCP-containing formulation restored flexural strength towards the value of unmodified PMMA. This result indicates that HEMA-mediated improvement of filler–matrix compatibility, rather than TCP alone, was a key determinant of the observed flexural response. In study [67], cement with 5% TCP showed lower compressive strength (~70 MPa) compared to unmodified cement (~85 MPa), but the values were within the ISO 5833 standards.

Study [68] evaluated TCP-based cements (KyphOs) doped with selenium nanoparticles. Pure TCP cement had lower compressive strength compared to PMMA, which is typical for ceramic materials. Modifications of PMMA with SeNPs reduced strength in proportion to the concentration of nanoparticles, while the addition of selenium did not significantly affect the mechanical properties of TCP itself, suggesting that partial amounts of TCP in the PMMA composition may, to some extent, mitigate the effect of cement modification with other components on mechanical strength.

Karpiński et al. reported that, in their tested PMMA system, 3 wt% α-TCP reduced compressive strength by approximately 8.5%, whereas β-TCP caused no statistically significant change in compressive strength up to 10 wt%. Shore hardness remained unchanged irrespective of the type and concentration of TCP [33].

It must be stressed, however, that this ≈10 wt% figure is not a universal ceiling but a condition-specific threshold, valid only for the particular combination of phase, microstructure and loading under which it was measured. Three qualifiers apply. First, phase: the absence of a statistically significant compressive-strength reduction up to 10 wt% was reported specifically for β-TCP in the solid PMMA formulation investigated by Karpiński et al. [33]. In the same study, α-TCP at 3 wt% reduced compressive strength by approximately 8.5%. These values should be interpreted as formulation- and test-specific observations, not universal compositional limits. Second, porosity: the value was established for essentially solid (low-porosity) specimens, in which the filler acts mainly as a diluent. Once porosity is deliberately introduced or generated by dissolution, the mechanical penalty is dominated by the pore population rather than by the nominal TCP fraction—porous PMMA/β-TCP falls to ≈50 MPa compressive strength and ≈900 MPa modulus, far below its dense counterpart at the same or lower ceramic content [60], so in a porous architecture the mechanically admissible β-TCP fraction is correspondingly lower. Third, loading regime: the ≈10 wt% threshold derives from quasi-static compression, the least demanding mode. Under the tensile, flexural and, above all, cyclic loading that dominates in vivo, the same microstructural defects that leave compressive strength almost unchanged act as crack-initiation and crack-growth sites, so fracture toughness and fatigue life degrade with rising porosity and filler content well before quasi-static compressive strength does [80,81,89]. The practical consequence is that the admissible TCP content should be read as a function—decreasing with α-phase fraction, with porosity and with the severity of cyclic loading—rather than as the single number “≤10 wt%”; the value quoted here should therefore be treated as an upper bound for solid, β-TCP-only cement under quasi-static compression, to be revised downward for porous, α-containing or fatigue-loaded formulations, and confirmed by the dynamic testing programme.

An extensive analysis of compressive strength, including both in vitro tests and long-term in vivo observations, was performed for Calcemex cement (PMMA + β-TCP) in a study by Maluta et al. One year after implantation in pig bones, the cement retained its original compressive strength, with minimal loss of mechanical parameters. The compressive strength of fresh, pre-polymerised Calcemex averaged 30.69 MPa, while after 12 months of in vivo use, this parameter decreased slightly to 29.24 MPa. For comparison, natural cancellous bone showed only 5.45 MPa, which emphasises that even after partial resorption of the mineral fraction, the cement retains sufficient stability for orthopaedic applications. After one year of use, Calcemex showed regularity of the fracture surface, with no premature fractures or decrease in resistance to crack propagation [69].

Cement G2B1 containing a total of 49.2% β-TCP showed significantly higher bone bonding strength than standard PMMA (CMW1) in all periods studied. These values ranged from 0.385 ± 0.199 MPa after 4 weeks to 0.858 ± 0.280 MPa after 12 weeks, which significantly exceeded the PMMA values ranging from 0.078 ± 0.095 MPa to 0.254 ± 0.110 MPa in the same periods [57].

Dall’Oca et al. also reported the high mechanical stability of these materials in a rabbit model for up to 12 months, although detailed compression test results were not presented [62].

Biomechanical comparisons of various materials for reinforcing pedicle screws showed that PMMA provided the highest pull-out strength, while HA/β-TCP (60% HA/40% β-TCP) exhibited better strength than the control sample, although lower than pure PMMA. The maximum pull-out force for the tested materials in descending order was as follows: PMMA, calcium sulphate, HA/β-TCP, allograft bone particles and the control sample [84]. These results suggest that although HA/β-TCP alone may not match PMMA in terms of strength, its use as an additive can improve the mechanical properties of cements.

In the case of copper-enriched tricalcium phosphate (Cu-TCP) admixture, it was observed that low concentrations (2.5% by weight) significantly increase the flexural modulus of PMMA. However, as the amount of Cu-TCP in the cement increases, the flexural modulus gradually decreases. A similar trend was observed for flexural strength. Interestingly, no significant effect of Cu-TCP particles on the compressive strength of PMMA cements was observed [66].

In the PMMA-TCP–chitosan formulations evaluated by Fang et al. [65], compressive strength, shear force, and elastic modulus decreased as the total TCP–chitosan phase increased. Because both the ceramic and polymeric co-additives changed simultaneously, the observed mechanical penalty cannot be assigned quantitatively to TCP alone. Pure PMMA had the highest compressive strength (127.4 ± 16.8 MPa), while biocomposites with increasing TCP admixture gradually obtained lower values of this property (from 109.0 ± 0.6 MPa for P3TC1 to 72.1 ± 6.2 MPa for P1TC3). Similarly, the shear force in cements decreased from 1266 N (pure PMMA) to approximately 800 N (highest TCP/chitosan content). With an increase in the proportion of TCP and chitosan in the composite, a decrease in Young’s modulus is observed—from 1278.3 MPa for pure PMMA to approximately 1019 MPa for the P1TC3 mixture. This means that these materials become less rigid. A lower Young’s modulus can potentially reduce the ‘stress-shielding’ effect—the uneven distribution of stress at the bone–cement interface, i.e., a situation where very rigid implants transfer too high a percentage of the load, leading to the loss of natural bone in the vicinity of the implant. With a lower Young’s modulus, the distribution of stresses in the adjacent bone tissue is more favourable for its preservation and remodelling, so potential limitations in mechanical strength in load-bearing applications should be taken into account [65].

In the porous PMMA-TCP formulation evaluated by Dall’Oca et al. [60], mechanical properties were substantially lower than in the non-porous comparator: compressive strength was approximately 50 MPa versus 100 MPa, flexural strength approximately 22 MPa versus 50 MPa, and flexural modulus approximately 900 MPa versus 3100 MPa. The porous formulation therefore did not meet the ISO 5833 minimum requirements for compressive strength (70 MPa) or flexural modulus (1800 MPa). Its porous architecture was associated with substantially improved bone affinity and tissue ingrowth, but this biological benefit was obtained at the cost of load-bearing capacity. Accordingly, this specific formulation should not be interpreted as suitable for conventional load-bearing cemented fixation without further formulation-specific mechanical validation. The addition of chitosan/β-TCP microspheres to PMMA cement also reduces compressive and flexural strength, which is a significant limitation in the use of these materials for mechanical load bearing [58].

The above results emphasise that TCP can both strengthen and weaken the mechanical properties of PMMA cements depending on the method and amount of its introduction, which makes the selection of TCP form and content the decisive design variable in this section.

A further limitation of the evidence surveyed above is that it is almost entirely quasi-static: compressive and flexural strengths and elastic moduli are single-point measurements taken shortly after setting, whereas an implanted cement mantle must sustain load for years. Two time-dependent phenomena therefore deserve explicit attention. The first is creep and stress relaxation. PMMA is a viscoelastic glassy polymer, so under the sustained physiological load of a cemented construct it undergoes slow, time-dependent deformation (creep) and, under fixed strain, a gradual decay of stress (relaxation); both were documented for acrylic bone cement decades ago and are strongly temperature- and stress-dependent [90]. Rigid, well-bonded ceramic particles generally raise the creep resistance of a polymer matrix by restricting chain mobility, so a dispersed TCP fraction may in principle reduce cement creep—an effect consistent with the higher macro- and nanoscale stiffness reported for particulate-reinforced cements [91]. However, this benefit is contingent on the same interfacial quality: where the TCP-PMMA bond is weak, or the filler is porous, particle-matrix sliding and pore compaction can instead accelerate creep and stress relaxation, promoting long-term subsidence and micromotion at the implant interface. For PMMA/TCP composites specifically, no dedicated creep or stress-relaxation data were identified in the reviewed literature, which is a clear gap given the direct relevance of these properties to aseptic loosening.

Another, and clinically more critical, phenomenon is the coupling of cyclic fatigue with hydrolytic ageing in body fluids. In vivo, the cement mantle is loaded not statically but cyclically—of the order of a million gait cycles per year—and fatigue failure nucleates at exactly the porosity and filler-related defects that quasi-static compressive strength is largely insensitive to [80,81]. Under physiologically relevant cyclic compression, fatigue life and fracture toughness are governed by interfacial bonding rather than by static strength [92]. Superimposed on this mechanical fatigue is chemical degradation: acrylic cement immersed in isotonic fluid at body temperature absorbs water by Fickian diffusion—accelerated by the vacancies left as residual monomer leaches out—which both plasticises the matrix and hydrolyses the outermost PMMA layers, progressively lowering its mechanical and fatigue properties over time [93]. TCP-modified formulations are expected to be more, not less, susceptible to this route: the hydrophilic ceramic and the interconnected porosity it creates raise water uptake, and the dissolution of the mineral phase both removes load-bearing material and opens fresh pathways for fluid ingress. This is borne out for closely related systems—chitosan/β-TCP-containing acrylic cements immersed in simulated body fluid showed markedly increased water uptake, mass loss and porosity over the immersion period [94]—and is fully consistent with the 2.6–12.8% mass loss in PBS over 60 days reported for the PMMA/TCP/chitosan cements [65]. The combined picture is therefore one of a material whose in-service mechanical performance is not the as-set value but a declining function of time, cyclic load history and fluid exposure—a durability dimension that the predominantly short-term, quasi-static literature has yet to quantify for PMMA/TCP cements, and which motivates the fatigue-in-SBF testing programme.

Taken together, the reviewed studies identify TCP phase and content, particle size, and pore structure as three interdependent variables that influence the bioactivity–mechanical-stability trade-off. The following ranges should be read as indicative, literature-derived observations, rather than as validated universal design rules, because they originate from heterogeneous formulations, test conditions, and biological models.

1. TCP content and polymorph (formulation-specific observations): Because the ceramic phase and its dissolution simultaneously drive osteoconduction and matrix weakening, the crystalline form should be matched to the intended balance. Where mechanical retention is the priority (e.g., endoprosthesis fixation), β-TCP is preferable and can be added up to approximately 10 wt%—a value that holds for solid cement under quasi-static compression and should be reduced for porous or fatigue-loaded constructs—without a statistically significant loss of quasi-static compressive strength or Shore hardness, whereas the more soluble α-TCP should be kept below approximately 3 wt%, since higher fractions reduce compressive strength by on average about 8.5%. These values are not universal design limits and should be re-evaluated for each PMMA base, particle size, porosity, co-additive system, ageing condition, and loading regime [33]. Where accelerated resorption and bone replacement are desired, higher β-TCP fractions (up to 40–70% within a biphasic β-TCP/HA phase) markedly enhance osteogenesis and in vivo bone formation while still meeting the ISO 5833 compressive-strength threshold, although the accompanying rise in particle brittleness caps the mechanically safe fraction [57,63].
2. Particle size (observations from porous animal-model formulations): Powder-scale fractions (~53 µm) generate only small, poorly connected pores and yield marginal remodelling, whereas granules in the 100–300 µm range open continuous channels that permit deep cell and vascular ingrowth and allow the resorption rate to be synchronised with new-bone formation; this fraction should therefore be regarded as the target range when biological integration is the design goal [60].
3. Pore structure (architecture reported in selected porous formulations): In the porous PMMA-TCP formulation reported by Dall’Oca et al. [60], micropores of approximately 10–15 μm and macropores of approximately 100–250 μm were associated with increased bone affinity and tissue ingrowth. However, this porous architecture imposed a substantial mechanical penalty: compressive strength was approximately 50 MPa and flexural modulus approximately 900 MPa, compared with approximately 100 MPa and 3100 MPa for the non-porous comparator. These porous-specimen values are below the ISO 5833 minimum requirements for compressive strength and flexural modulus and therefore should not be presented as compliant with the requirements for conventional load-bearing bone cement. This formulation illustrates the biological benefit–mechanical cost of deliberately introduced porosity; whether a porous PMMA-TCP system is suitable for a particular application must be determined from its own standardised mechanical, fatigue, and in vivo performance data.

**Figure 4:**
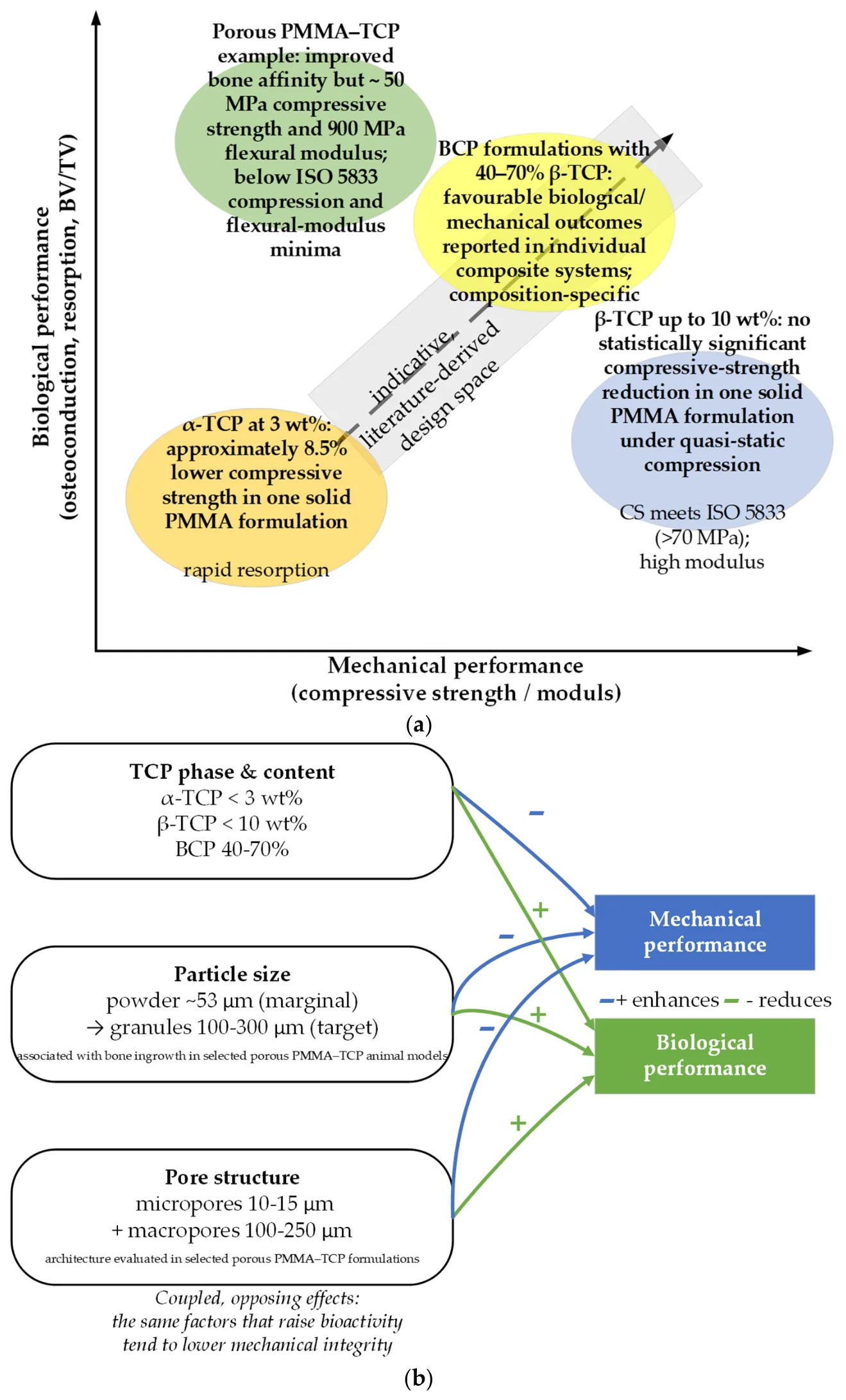
Qualitative synthesis of the bioactivity–mechanical-stability trade-off in PMMA–TCP bone–cement formulations: (**a**) conceptual relationship between mechanical and biological performance; (**b**) influence of TCP phase/content, particle size, and pore structure.

Across the selected studies, moderate β-TCP loading in a solid PMMA formulation, larger TCP granules in porous formulations, and interconnected micro- and macroporosity were each associated with a more favourable balance between biological response and short-term mechanical performance. However, these observations derive from individual formulations tested under different experimental conditions and are not sufficient to define a universal optimum composition or pore architecture. Figure 4 therefore provides a qualitative, literature-derived illustration of the opposing mechanical and biological effects of TCP phase/content, particle size, and pore structure. The positions shown in the mechanical–biological trade-off plane represent selected example formulations from the reviewed studies, rather than pooled data, validated performance boundaries, or universally applicable design recommendations.

Overall, the evidence that compressive strength declines with increasing TCP content, and that α-TCP is more detrimental than β-TCP at comparable loading, is consistent in direction and may be regarded as of moderate certainty. The specific concentration thresholds cited above, however, derive from a single graded concentration series and are of limited certainty pending independent replication. The finding that deliberately porous PMMA-TCP formulations fall below the ISO 5833 minima is likewise supported by a single study and is therefore of limited certainty, although it is consistent with the expected effect of porosity on compressive behaviour.

### 3.5. Change in Operating Characteristics

PMMA cements containing TCP also exhibit other beneficial performance properties that are important for clinical applications. The addition of TCP affects the setting time of the cement. The G2B1 composition with 49.2% β-TCP in its composition had an appropriate setting time, and the working time window (the difference between the initial and final setting times) was approximately five minutes, which was comparable to six minutes for PMMA [57]. For composites with chitosan/β-TCP microspheres, the setting time increases from 3.5 to 9 min compared to pure PMMA cement [58]. Studies conducted by Cojocaru and colleagues showed that the addition of chitosan/TCP as a component to PMMA cement prolonged the setting time from 4 to 7 min compared to PMMA cement [61].

In PMMA composites containing both TCP and chitosan, setting time increased with increasing content of the combined additive phase. The relative contributions of TCP, chitosan, altered powder-to-monomer ratio, and porosity were not independently resolved. Introduction of TCP and chitosan into the PMMA matrix significantly prolongs the setting time of the cement—from 7.5 min (pure PMMA) to as long as 15 min (highest additive content). The longer setting time gives the operator greater freedom and safety when applying the cement during the procedure, reducing the risk of premature setting and minimising the risk of incorrect implantation [65].

One of the significant problems associated with PMMA bone cements is the high temperature generated during polymerisation, which can lead to necrosis of surrounding tissues. Studies have shown that the addition of chitosan/TCP and chitosan/HA to PMMA cement reduces the peak curing temperature [61]. This is a very beneficial effect, as it contributes to reducing thermal damage to surrounding tissues during cement application. In the study by [64], TCP and HEMA cements maintained a setting time (~770 s) and polymerisation temperature (~80 °C) similar to PMMA, meeting the requirements of ISO 5833. In study [67], the addition of TCP did not significantly affect these parameters. The ptcpPMMA cement showed a lower maximum in vivo polymerisation temperature (average 33.68 °C) than pPMMA (35.15 °C), which reduces the risk of thermal necrosis of adjacent tissues. The difference was due to the lower PMMA content in ptcpPMMA [59]. It was determined that porous PMMA/β-TCP cement has a polymerisation temperature of 44 °C compared to 70 °C for standard PMMA cement [60]. Similar results were observed when chitosan/β-TCP microspheres were added to PMMA cement [58]. In another study of TCP-modified cements, the maximum temperature reached during exothermic curing was reduced—for pure PMMA it was 72 °C, while for TCP-modified cements (P1TC3: lowest PMMA content, highest amount of TCP + chitosan phase = 1:3), it was reduced to 43.5 °C [65].

Analysis of injectable biphasic calcium phosphate composite cements (in the form of a mixture of HA and β TCP) BCPx/PMMA with varying contents of the biphasic calcium phosphate phase showed that such cement is an injectable material and that the developed composites are suitable for vertebroplasty applications, which implies an appropriate range of viscosity/fluidity of the material during operation and an acceptable setting time [63].

The last parameter analysed was wettability, a factor determining how the material interacts with body fluids and cells, and thus indirectly with cement-bone integration. Changes in the composition of the cement caused modifications in the nature of the structure. The authors used contact angle measurements as a simple, quantitative indicator of the extent to which the added biodegradable components can improve the biological surface (degree of hydrophilicity), which was reflected in the wetting angle values and correlated with the cellular response described in the study (adhesion, proliferation, and cell metabolism friendliness of the cement), without significantly impairing its mechanical parameters. It was shown that TCP increased the contact angle (hydrophobicity) from ~65° (pure PMMA) to ~123°, which, however, may hinder cell adhesion [67].

In terms of performance, it has been shown that TCP additives (often in combination with chitosan or other biopolymers) prolong the setting time of cement, improving operator comfort and reducing the peak polymerisation temperature from approx. 70–80 °C for classic PMMA to around 40–45 °C in porous composites, which significantly reduces the risk of thermal necrosis of surrounding tissues [95]. Attention has also been drawn to the possibility of modulating wettability and surface characteristics (contact angle, hydrophilicity/hydrophobicity) by selecting the type of additives, which translates into cell adhesion and osseointegration potential, although too strong an increase in hydrophobicity by TCP may hinder cell colonisation of the surface. Studies also indicate that combining TCP with metal nanoparticles (AgNPs, SeNPs, and Cu-TCP) can give cements antibacterial properties while maintaining acceptable mechanical parameters and low cytotoxicity [96]. A normalised, cross-study comparison of all reviewed PMMA/TCP formulations along these unified parameters-base system, TCP phase, content, particle size, porosity, testing method/standard, study type, biological model and key quantitative outcomes-is compiled in Table 1, which is intended to support direct, like-for-like interpretation of the heterogeneous data discussed above.

As the dedicated study-type column in Table 1 makes explicit, the current evidence rests predominantly on in vitro and short-term in vivo animal studies, with only a few formulations assessed under combined in vitro and in vivo conditions, underlining the need for cautious clinical extrapolation.

The evidence concerning setting behaviour and peak exotherm is of limited certainty, resting on only a few studies, several of which report the direction of change without the corresponding values.

The most important gaps and limitations identified in the review mainly concern the nature and scope of available mechanical tests. Short-term static tests (compression, bending, hardness) conducted in vitro, often under idealised conditions, with manual mixing of cement and limited porosity control, dominate, which the authors themselves point to as a source of additional structural defects and variability in results [97]. There is a lack of systematic fatigue testing under cyclic loading conditions, creep and stress relaxation testing in physiological fluids, and analyses of mechanical relationships to real-world loading patterns in different bone locations (spine, hip, knee), which makes it difficult to translate the results into long-term clinical use [98].

Another limitation is the significant heterogeneity of the formulations used (different PMMA bases, additives, TCP grain sizes, different animal models), which makes direct comparisons and the construction of consistent composition–microstructure–property maps difficult [97]. It is also worth noting that few studies directly compare the mechanical cost of bioactivity (loss of strength, decrease in modulus) with measurable osseointegration benefits in the same research configuration [65]. In this context, the deliberately limited sample of fifteen studies should be understood as a methodological choice favouring thematic relevance and comparability over sheer number, rather than as a deficiency of the review.

A further limitation is that six reports could not be included because a sufficiently complete full text could not be obtained despite retrieval attempts through institutional subscriptions and library resources, publisher open-access platforms, author-shared manuscripts, and direct full-text requests through ResearchGate. This may have introduced accessibility-related selection bias and may mean that some relevant evidence is under-represented in the final corpus. Therefore, the risk of bias due to missing results was evaluated separately for each of the three syntheses.

The mechanical synthesis was the most affected by selective non-reporting. Several included reports described the direction of change in compressive or flexural behaviour without providing the corresponding values, reported means without any measure of dispersion, or presented composite figures from which individual group means could not be recovered; the affected outcomes are identified in Table 2. The quantitative conclusions of this synthesis therefore rest on a smaller number of reports than the number of reports contributing to it, and the reported ranges of strength should be read as ranges of the values that were disclosed rather than of the values that were measured.

The biological synthesis was affected to a lesser degree, the reported outcomes being predominantly quantitative, but measures of dispersion and stated specimen numbers were frequently absent, and one study reported osteointegration only on a semi-quantitative histological scale. Follow-up durations were reported inconsistently, which limits the extent to which time-dependent behaviour can be compared across studies.

The synthesis of handling and functional characteristics rests on the smallest number of studies and is consequently the most vulnerable to missing results, whether unreported within the included studies or absent because a potentially eligible report could not be obtained. Peak exotherm and setting time were in some cases reported only as reduced or prolonged relative to the unmodified control, without values.

At the level of the corpus, 5 of the 20 reports assessed as potentially eligible could not be obtained in full text, corresponding to ¼ of that set. Their outcomes are unknown, and their absence may affect any of the three syntheses. It should be noted, however, that these reports were unavailable predominantly because of paywall restrictions, a mechanism that is not expected to be related to the direction or magnitude of the results they contain. The resulting risk is therefore one of reduced precision rather than of directional bias. A distinct consideration arises from the eligibility criteria themselves: conference abstracts and reports not presenting primary experimental data were excluded, and since unfavourable results in materials research are disproportionately reported in such formats, formulations that failed to perform as intended may be under-represented in the corpus. Because no quantitative synthesis was undertaken, none of these effects can be estimated, and all three syntheses should be read as descriptions of the reported evidence rather than of all evidence generated.

It should be emphasised that the corpus of fifteen studies is a direct and intended consequence of the selection strategy adopted, rather than a shortcoming of the review. The corpus was deliberately restricted to primary experimental works that simultaneously satisfy three demanding criteria: they concern PMMA-based cements specifically modified with tricalcium phosphate (and not calcium-phosphate or other ceramic systems in general), they report quantitative data on the mechanical, biological or handling properties relevant to this review, and they fall within the fifteen-year window that already covers roughly two-thirds of the entire literature on PMMA bone cements. As shown in Figure 1 and Figure 2, the number of publications addressing the narrow “bone cement AND pmma AND (tcp OR “tricalcium phosphate”)” topic became significant only after 2000 and remains modest in absolute terms; consequently, fifteen studies that meet all inclusion criteria constitute a representative rather than a marginal sample of the directly relevant evidence. Precedence was therefore given to the thematic relevance and methodological comparability of the included works over their sheer number.

Artificially inflating this figure by reaching back to older publications would, in our view, weaken rather than strengthen the review. First, the formulations, TCP grades and particle-size ranges used in earlier decades differ substantially from those employed today, so their mechanical and biological results are not directly comparable and would introduce heterogeneity that obscures, rather than clarifies, the composition–microstructure–property relationships this review seeks to establish. Second, the experimental methods and instrumentation used to characterise these cements—standardised mechanical testing regimes, imaging techniques such as micro-CT and SEM, and in vivo evaluation protocols—have evolved considerably, and data obtained with outdated apparatus or non-current testing conventions cannot be pooled with contemporary measurements without a serious risk of bias. Third, a large fraction of the older records would duplicate, in updated form, findings already captured by the recent studies, adding volume without adding evidential value. For these reasons, expanding the sample solely to increase the number of analysed papers would compromise the internal consistency and the clinical relevance of the synthesis; a focused corpus of methodologically comparable, up-to-date studies provides a sounder basis for the conclusions drawn here.

Beyond the mechanical domain, several further limitations temper the strength of the current evidence base and should guide its interpretation:

1. The body of work is dominated by in vitro and short-term assessments, while long-term in vivo data and, above all, controlled clinical studies with implant-survival endpoints remain scarce; because in vitro results are frequently not representative of the in vivo situation, the direct clinical translation of the reported mechanical and biological benefits is still uncertain [86]. This is compounded by the reliance on heterogeneous animal models, whose bone architecture, remodelling rate and healing capacity differ from those of humans, which limits the extent to which osseointegration outcomes can be extrapolated to clinical practice [99].
2. The mechanical and setting data reported across studies are not always acquired under fully harmonised, standard-compliant conditions (e.g., ISO 5833/ASTM F451), with variations in specimen geometry, loading rate and conditioning medium that further reduce inter-study comparability and can, in themselves, produce statistically significant differences in the measured properties [31].
3. Information on the long-term durability of PMMA/TCP composites is limited: the progressive dissolution and resorption of the mineral phase inevitably increases porosity over time, yet its consequences for fatigue life and structural integrity over clinically relevant periods have rarely been quantified [31].
4. The functional and handling characteristics that are decisive at the point of surgery, such as mixing method, injectability and working time, are often reported qualitatively and without standardised protocols, even though poor or manual handling is known to impair the cement-implant interface and the reproducibility of the final microstructure [100].
5. The evidence may be affected by a degree of publication bias towards positive findings, by generally small sample sizes within individual studies, and by the near-absence of meta-analyses, all of which call for cautious, qualitative rather than firmly quantitative conclusions at the present stage.

Further research in the mechanical field should focus on the dynamic characteristics of PMMA/TCP cements under conditions that reflect clinical loads as closely as possible. The natural next step is to design a series of compression and flexural fatigue tests on samples with controlled α- and β-TCP content (e.g., 0–10% *w*/*w*) and strictly controlled porosity, conducted in a simulated body fluid (SBF, PBS) environment at 37 °C, with recording of parameter changes to subthreshold damage levels. A valuable addition would be dynamic step-loading or block-loading tests, mimicking variable gait cycles and daily activities, with simultaneous monitoring of the microstructure (micro-CT and digital image correlation) over time, which would allow the initiation and propagation of microcracks to be linked to the distribution and degradation of the TCP phase. It also seems reasonable to use biomechanical models of bone segments (e.g., vertebral bodies and long bone epiphyses) filled with PMMA/TCP and perform quasi-static and dynamic tests (compression, screw extraction, torsion) with recording of structural stiffness, energy absorbed to destruction and local deformations in bone.

It is important to consider impact and quasi-dynamic tests (Charpy/Izod, drop-weight) to evaluate the behaviour of modified cements under short-term overloads, such as falls or sudden load changes, which can be critical in osteoporotic vertebrae or in the area of endoprosthesis stems. Another interesting direction is the assessment of the coupling of dynamic loads with TCP degradation and leaching, i.e., tests in which PMMA/TCP samples are subjected to long-term cyclic loading in a fluid environment, with periodic analysis of the chemical composition of the solution (Ca^2+^, PO_4_^3−^ ion release) and changes in mechanical properties and pore morphology. In addition, push-out and pull-out fatigue tests can be introduced in bone models with PMMA/TCP cements to quantitatively link the increase in osseointegration with the durability of the connection under cyclic loading, using numerical modelling (FEA) in parallel to assess stress distribution and the stress-shielding effect.

A mechanical testing programme constructed in this way would fill the gaps identified in the review and move from a qualitative description of the advantages of TCP to a quantitative, engineering design of PMMA/TCP formulations for specific clinical load scenarios.

The bioactivity–mechanical-stability trade-off in PMMA/TCP bone cements is presented in synthetic form in Figure 4: a trade-off map positioning typical formulation classes on the mechanical versus biological performance axes, with the optimal design window highlighted and influence diagram linking the three principal design variables—TCP phase/content, particle size and pore structure—to mechanical and biological performance, where (+)/(−) signs denote the coupled, opposing effects. Thresholds are derived from the data compiled in Table 1 and Table 2 and from refs. [33,57,60,63]. The α-TCP and β-TCP content annotations are based on the specific solid PMMA formulation and quasi-static compressive testing conditions reported by Karpiński et al. [33]. Their applicability depends on the PMMA base, TCP polymorph and morphology, total ceramic loading, porosity, co-additives, processing method, conditioning, and loading regime.

Setting aside the reports that present previously published data did not change the direction of any conclusion, since the findings concerned were corroborated by at least one independent primary study, although the quantitative support for the effect of TCP on setting behaviour was reduced. Setting aside the studies in which TCP cements were assessed alongside rather than within a PMMA matrix likewise left the direction of all conclusions unchanged, but narrowed the range of reported compressive strengths substantially, from approximately 2.6–237 MPa to approximately 50–146 MPa; the wider range should therefore not be read as the range attainable by TCP-modified PMMA. Setting aside the study co-authored by the present authors had the greatest effect: the qualitative conclusion that compressive strength declines progressively with increasing TCP content, and that α-TCP is more detrimental than β-TCP at equal loading, remained supported, but the specific quantitative thresholds no longer had independent quantitative support, since no other included study reported a graded concentration series for both polymorphs under a single test protocol. These thresholds should accordingly be regarded as provisional and in need of independent replication. Excluding values read from figures did not alter the direction of any finding, but removed the numerical basis for the reported effect of HEMA content on compressive strength.

The evidence that TCP-containing formulations exhibit prolonged setting and reduced peak exotherm is consistent in direction and of moderate certainty, whereas the magnitude of these changes is of limited certainty; several reports describe the direction without reporting values, and the changes cannot be attributed to TCP alone where powder-to-monomer ratio and co-additives varied simultaneously.

## 4. Conclusions

This review indicates that incorporation of tricalcium phosphate (TCP) into PMMA-based bone–cement formulations can introduce a resorbable calcium-phosphate phase and can contribute to changes in microstructure, osteoconductive potential, and mechanical behaviour. The direction and magnitude of these effects depend on TCP polymorph, content, particle size, pore structure, PMMA base formulation, and loading conditions. The most direct TCP-specific mechanical evidence derives from otherwise comparable PMMA formulations in which TCP type or concentration was varied, showing that β-TCP can be incorporated at moderate contents without a statistically significant reduction in quasi-static compressive strength, whereas higher fractions of the more soluble α-TCP may reduce strength. In contrast, findings from formulations containing additional modifiers, including HA/BCP, HEMA, chitosan, AgNPs, SeNPs, Cu-containing phases, or bioactive glass, should be interpreted as properties of the complete composite rather than as effects attributable to TCP alone. Porous PMMA-TCP formulations may improve bone ingrowth and osteoconductive potential, but the porous system reported by Dall’Oca et al. [60] showed compressive strength of approximately 50 MPa and flexural modulus of approximately 900 MPa, below the respective ISO 5833 minimum requirements. Such formulations should therefore be regarded as biologically oriented, mechanically compromised materials whose use in load-bearing cemented fixation cannot be inferred from the available evidence. In particular, the reported antibacterial activity of Ag-, Se-, and Cu-containing systems is primarily associated with these antimicrobial phases. Although preclinical studies indicate potential for improved bone interaction and remodelling in selected formulations, the evidence remains heterogeneous and predominantly limited to in vitro and animal models; therefore, clinical performance, long-term durability, and applicability to specific indications, including osteoporotic bone, require further validation. All biological and handling-related benefits discussed above remain preclinical observations and should not be interpreted as evidence of improved clinical fixation, reduced thermal injury, or superior patient outcomes.

Several TCP-containing PMMA composite formulations, particularly porous systems and those additionally containing chitosan or other co-modifiers, showed longer setting times and lower peak polymerisation temperatures than their reference PMMA cements. These handling and thermal effects are formulation-specific and may reflect not only TCP loading but also reduced PMMA content, altered powder-to-monomer ratio, porosity, and the presence of co-additives. Accordingly, the available evidence does not support attributing these changes universally to TCP alone. Additional modifications, such as chitosan, silver nanoparticles, selenium or Cu-TCP, allow the materials to be given antibacterial functionality and modulate surface wettability, but require further verification of their long-term impact on strength and biological safety.

In practical terms, the reviewed evidence points towards a provisional set of design considerations rather than validated design rules. The direction of the underlying relationships—that increasing TCP content reduces compressive strength, that α-TCP is more detrimental than β-TCP, and that increasing porosity trades mechanical performance for bioactivity—is consistently supported. The numerical values that follow, by contrast, derive from single studies and are offered as starting points for further investigation rather than as established limits: an upper bound of approximately 10 wt% β-TCP for solid cement under quasi-static compression, to be lowered for porous or cyclically loaded constructs, granules in the 100–300 µm range, a bimodal micro-/macroporous architecture of approximately 10–15 µm and 100–250 µm and avoidance of α-TCP contents above approximately 3 wt% in load-bearing applications. None of these values has been independently replicated under a common test protocol.

The current evidence base is predominantly preclinical and consists of in vitro material and cell-culture studies together with animal experiments; no controlled clinical trials or long-term patient follow-up studies of TCP-modified PMMA bone cements were identified. Consequently, reported reductions in peak polymerisation temperature, changes in handling behaviour, improved cell-related outcomes, bone ingrowth, and osteointegration should be interpreted as experimental findings rather than as demonstrated clinical benefits. In particular, a lower exothermic peak in a laboratory or animal model does not establish a reduction in thermal necrosis in patients; evidence of bone ingrowth does not establish improved long-term implant fixation; and short-term quasi-static mechanical results do not establish suitability for load-bearing clinical applications. Future work should include standardised mechanical testing, long-term fatigue, creep, and hydrolytic-ageing experiments under physiologically relevant conditions, followed by clinically relevant animal studies and controlled clinical investigations with safety, fixation, implant-survival, and patient-reported outcomes.

## Data Availability

No new data were created or analysed in this study. Data sharing is not applicable to this article.

## Supplementary Materials

The following supporting information can be downloaded at: https://www.mdpi.com/article/10.3390/ma19173772/s1, PRISMA 2020 checklist.
